# Sodium-calcium exchanger-3 regulates pain “wind-up”: From human psychophysics to spinal mechanisms

**DOI:** 10.1016/j.neuron.2022.05.017

**Published:** 2022-06-14

**Authors:** Teodora Trendafilova, Kaustubh Adhikari, Annina B. Schmid, Ryan Patel, Erika Polgár, Kim I. Chisholm, Steven J. Middleton, Kieran Boyle, Allen C. Dickie, Evangelia Semizoglou, Jimena Perez-Sanchez, Andrew M. Bell, Luis Miguel Ramirez-Aristeguieta, Samar Khoury, Aleksandar Ivanov, Hendrik Wildner, Eleanor Ferris, Juan-Camilo Chacón-Duque, Sophie Sokolow, Mohamed A. Saad Boghdady, Andre Herchuelz, Pierre Faux, Giovanni Poletti, Carla Gallo, Francisco Rothhammer, Gabriel Bedoya, Hanns Ulrich Zeilhofer, Luda Diatchenko, Stephen B. McMahon, Andrew J. Todd, Anthony H. Dickenson, Andres Ruiz-Linares, David L. Bennett

**Affiliations:** 1 Nuffield Department of Clinical Neurosciences, Oxford University, Oxford, UK; 2 School of Mathematics and Statistics, Faculty of Science, Technology, Engineering and Mathematics, The Open University, Milton Keynes, UK; 3 Department of Genetics, Evolution and Environment, University College London, London, UK; 4 Department of Cell and Developmental Biology, University College London, London, UK; 5 Department of Neuroscience, Physiology and Pharmacology, University College London, London, UK; 6 School of Psychology and Neuroscience, University of Glasgow, Glasgow, UK; 7 Wolfson Centre for Age-Related Diseases, King’s College London, London, UK; 8 QST Lab. Faculty of Odontology, Universidad de Antioquia, Medellin, Colombia; 9 McGill University and Genome Quebec Innovation Centre, Montreal, QC, Canada; 10 Department of Physiology Anatomy and Genetics, University of Oxford, Oxford, UK; 11 Institute of Pharmacology and Toxicology, University of Zurich, Zurich, Switzerland; 12 Centre for Palaeogenetics, Stockholm, Sweden; 13 Department of Archaeology and Classical Studies, Stockholm University, Stockholm, Sweden; 14 Laboratoire de Pharmacodynamie et de Thérapeutique Faculte de Médecine Université Libre de Bruxelles, Brussels, Belgium; 15 School of Nursing, University of California, Los Angeles, Los Angeles, CA, USA; 16 CNRS, EFS, ADES, Aix-Marseille Université, Marseille, France; 17 Unidad de Neurobiologia Molecular y Genetica, Laboratorios de Investigación y Desarrollo, Facultad de Ciencias y Filosofía, Universidad Peruana Cayetano Heredia, Lima, Peru; 18 Instituto de Alta Investigacion, Universidad de Tarapaca, Arica, Chile; 19 GENMOL (Genetica Molecular), Universidad de Antioquia, Medellin, Colombia; 20 Institute of Pharmaceutical Sciences, Swiss Federal Institute of Technology (ETH) Zurich, Zurich, Switzerland; 21 Ministry of Education Key Laboratory of Contemporary Anthropology and Collaborative Innovation Center of Genetics and Development, School of Life Sciences and Human Phenome Institute, Fudan University, Shanghai, China

## Abstract

Repeated application of noxious stimuli leads to a progressively increased pain perception; this temporal summation is enhanced in and predictive of clinical pain disorders. Its electrophysiological correlate is “wind-up,” in which dorsal horn spinal neurons increase their response to repeated nociceptor stimulation. To understand the genetic basis of temporal summation, we undertook a GWAS of wind-up in healthy human volunteers and found significant association with *SLC8A3* encoding sodium-calcium exchanger type 3 (NCX3). *NCX3* was expressed in mouse dorsal horn neurons, and mice lacking *NCX3* showed normal, acute pain but hypersensitivity to the second phase of the formalin test and chronic constriction injury. Dorsal horn neurons lacking *NCX3* showed increased intracellular calcium following repetitive stimulation, slowed calcium clearance, and increased wind-up. Moreover, virally mediated enhanced spinal expression of *NCX3* reduced central sensitization. Our study highlights Ca^2+^ efflux as a pathway underlying temporal summation and persistent pain, which may be amenable to therapeutic targeting.

## Introduction

In humans, repetitive or prolonged noxious stimulation results in increased pain perception, a psychophysical phenomenon termed temporal summation (Arendt-Nielsen, 2015). This is frequency dependent; occurs following the application of high-threshold mechanical (Koltzenburg and Handwerker, 1994), electrical (Lundberg et al., 1992), or thermal stimuli (Vierck et al., 1997); and can be elicited from skin, musculoskeletal, and visceral tissues. Electrophysiological assessment of nociceptor activity and also the withdrawal reflex in humans have shown that such temporal summation is due to activity-dependent plasticity within the central nervous system (CNS) (Arendt-Nielsen et al., 1994; Koltzenburg and Handwerker, 1994). An important neural correlate of this temporal summation of pain is the phenomenon of “wind-up” of the responses of dorsal horn (DH) neurons within the spinal cord. Wind-up refers to the progressively increased response of predominantly deep DH neurons over the course of a train of repetitive C-fiber nociceptor stimulation (Mendell and Wall, 1965); this is frequency dependent, occurs when stimuli are delivered between 0.5 and 5 Hz, and requires C-fiber activation (there is no wind-up in response to stimulation of A-fibers). Wind-up has been reported in rodent (Dickenson and Sullivan, 1987; Sivilotti et al., 1993), cat (Mendell and Wall, 1965), and primate DH (Chung et al., 1979) and is one of the neural mechanisms underlying central sensitization (the increased responsiveness of nociceptive neurons in the CNS to their normal or subthreshold afferent input). The synaptic basis of wind-up is that the activation of peptidergic nociceptors projecting to DH neurons results in the co-release of calcitonin gene-related peptide (CGRP) and substance P along with glutamate (D’Mello and Dickenson, 2008; Latremoliere and Woolf, 2009). These neuropeptides activate CGRP1 and NK1 receptors, expressed by DH neurons, evoking slow synaptic potentials that summate to produce progressive membrane depolarization. This depolarization enables the activation of NMDA receptors due to the removal of voltage-dependent Mg^2+^ block, amplifying depolarization and the activity of DH neurons (Dickenson and Sullivan, 1987; Sivilotti et al., 1993). Increased intracellular Ca^2+^ following the activation of NMDA receptors can further impact synaptic signaling, sensitizing the NMDA receptor even more (Chen and Huang, 1992). The NMDA receptor has also been implicated in the temporal summation of pain in humans (Arendt-Nielsen, 2015). In addition to these synaptic changes, there are also circuit-level properties within the DH that mediate less pronounced wind-up of spinoparabrachial projection neurons (Hachisuka et al., 2018).

The recent standardization of quantitative sensory testing protocols to assess the psychophysical response (the relationship between sensory percept and stimulus) to sensory stimuli in humans and apply them to larger cohorts are improving our understanding of the inter-individual variation in pain perception. Twin studies have shown a significant heritability of these evoked pain phenotypes (up to 50%), depending on the modality (Nielsen et al., 2012). Determining the genetic basis of these pain phenotypes in humans could provide a fundamental understanding of the neurobiology of pain and potentially also identify novel treatment targets. A number of these experimental pain measures, including temporal summation, have been shown to be enhanced in clinical disorders such as musculoskeletal, visceral, and neuropathic chronic pain (Arendt-Nielsen, 2015). Furthermore, temporal summation has been reported to be predictive of clinical disorders such as post-surgical pain (Weissman-Fogel et al., 2009). There have been a number of candidate gene studies applied to quantitative sensory testing, including temporal summation (Sachau et al., 2019), but many of these findings have yet to be replicated. Besides, there have been no genome-wide association studies (GWASs) reporting significant associations with experimental pain traits. Here, we performed a GWAS for temporal summation of pain (expressed as wind-up ratio [WUR]) in a sample of ~1,000 healthy volunteers of mixed European/Native American/African ancestry, which was then replicated in a further ~300. WUR demonstrated genome-wide significant association with the SLC8A3 locus encoding the sodium-calcium exchanger type 3 (NCX3), which we then validated in mouse models demonstrating that this is a critical determinant of calcium handling and the activity-dependent plasticity of DH neurons.

## Results

### Human genetics analysis shows significant association between WUR and *NCX3*

**Figure 1:**
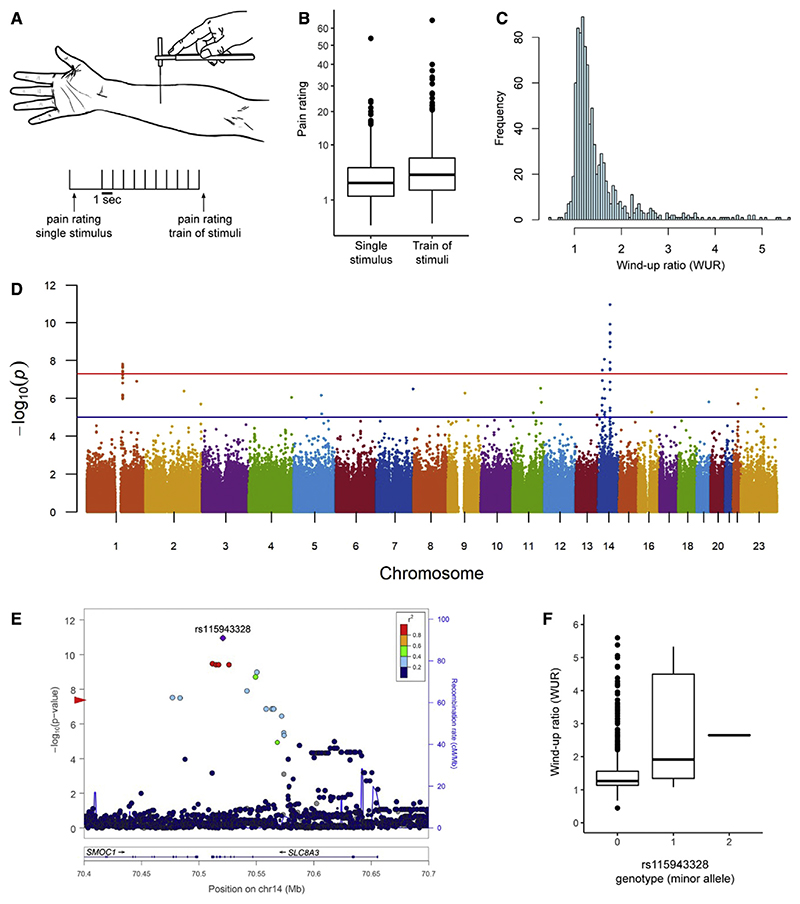
Clinical wind-up ratio is associated with *SLC8A3/NCX3* (A) Clinical wind-up ratio (WUR) was performed over the mid ventral forearm using a 255-mN von Frey hair and recording a pain rating for a single stimulus followed by a series of 10 stimuli. (B) Boxplots of numerical pain ratings for the single stimulus and the train of 10 stimuli. (C) Frequency histogram of the WUR. (D) Manhattan plot of the GWAS results of WUR. The red and blue lines indicate genome-wide and suggestive p value significance thresholds, respectively. (E) LocusZoom plot of the association results around the index SNP rs115943328 in *SLC8A3* (encoding *NCX3)*. The red arrow on the y axis indicates the GWAS genome-wide significance threshold. (F) Boxplot of WUR values against the three genotype categories of rs115943328 (corresponding to the minor allele C).

We obtained the WUR as a measure of temporal summation in 1,061 healthy individuals, recruited in Medellín (Colombia), with a median age of 23 (range 18–45) years, of which 55% were female. WUR was evaluated using a validated protocol stimulating with a 255-mN von Frey hair (Touch Test, North Coast, USA). Pain ratings were recorded on a numerical scale (0–100) for a single stimulus applied to the forearm, which were then followed by a train of 10 stimuli applied at 1 Hz in the same 1-cm^2^ area as the single stimulus, and the average was recorded. This procedure was repeated five times, and WUR was calculated as the mean pain rating for the train of 10 stimuli divided by the mean of the single stimulus (Rolke et al., 2006; Schmid et al., 2019). After quality control (see STAR Methods), the median pain rating for the single stimulus was 2.85 (interquartile range [IQR] 3.85) and 4.00 (IQR 5.14) for the train of stimuli (Figure 1B, this increase was highly statistically significant, Wilcoxon signed-rank p value < 1E–16). The median WUR was 1.27 (IQR 0.46), with the trait distribution having a longer tail toward high values (Figure 1C).

Individuals were genotyped on Illumina’s OmniExpress chip containing 730,525 SNPs across the genome, of which 673,034 SNPs were retained after applying quality control filters (see STAR Methods). We performed genotype imputation based on 1,000 Genomes data, resulting in a final genetic dataset that includes 9,616,058 autosomal and X chromosome SNPs. After quality control of the data for individuals, 991 subjects were retained for further analyses.

Based on the genome-wide SNP data, the average ancestry of these individuals was estimated as 29% Native American, 61% European, and 10% African (Figure S1A). Women were observed to have a small but significantly greater WUR, relative to men (1.51 versus 1.40; Spearman’s p value 1.3 x 10^−7^). Native American ancestry and self-reported depression score had a low but significant correlation with WUR (Pearson’s r = 0.06, p value 1 X 10^−2^ and r = 0.05, p value 2 x 10^−2^, respectively). Nosignificant effect for age was observed.

Association testing detected three clusters of SNPs, in 1q21.3, 14q24.2, and 14q21.2, exceeding the threshold for genome-wide significance (p value < 5.3 x 10 ^−8^; Figure 1; Table S1). The SNPs in 1q21.3 are intergenic but closest to S100A16(Figure S1B) encoding an EF-hand containing calcium-binding protein (Figure S1B). The SNPs in 14q24.2 overlap the *SCL8A3/NCX3* gene (encoding the Na^+^/Ca^2+^ exchanger 3) (Figure 1E), and those in 14q21.2 overlap *LINC00871* (long intergenic non-protein-coding RNA 871) (Figure S1C). These SNPs have a low polymorphism in Europeans and Native Americans, with minor allele frequencies >10% being observed only in Africans, and explain 3%–5% of the phenotypic variance (Table S1 shows the values for index SNPs in each region, i.e., those with the smallest p value). Consistent with their admixed ancestry, minor allele frequencies at these SNPs in the Colombians examined are intermediate between Europeans/Native Americans and Africans (Table S1).

We evaluated replication of the GWAS signals observed in the Colombian sample in two different ways. First, we examined association for index SNPs (those with the smallest GWAS p value) at each of the three genome regions in a second Colombian cohort (n = 317), phenotyped and genotyped in the same way as the initial cohort (Table S1). All three index SNPs showed nominally significant association (p value < 0.016) in this second Colombian cohort, with the strongest signal being observed for rs115943328 in *NCX3* (p value 3 × 10^−5^). Additionally, we analyzed data from the orofacial pain prospective evaluation and risk assessment (OPPERA) study cohort, which has also been characterized for a temporal summation phenotype (Kim et al., 2019). This cohort was recruited in the USA and is genetically highly heterogeneous (and differentiated) from the primary Colombian cohort. We therefore analyzed the OPPERA data using a gene-level association test. We found strongly significant association for *NCX3* (p value 3 × 10^−8^) and weakly significant association for LINC00871 (p value 0.005) (Table S1). The gene-level test did not show significant association for S100A16 (Table S1) in the OPPERA cohort.

ENCODE RNA sequencing (RNA-seq) data indicate maximal expression of *NCX3* in the spinal cord (ENCODE Project Consortium, 2012), whereas GTex data show that NCX3 is widely expressed in the human CNS (key resources table) (The GTEx Consortium, 2020). The associated SNPs in 1q21.3 have been identified as expression Quantitative Trait Loci (eQTLs) for S100A16 in the GTex data (The GTEx Consortium, 2020). The GTex data also show that S100A16 is expressed in human CNS (key resources table) (The GTEx Consortium, 2020), although with relatively low expression in mouse DH (Zeisel et al., 2018). The association of *NCX3* SNPs with WUR, the replication of this association in independent Colombian and North American cohorts, and the reported expression of *NCX3* in the human CNS (including spinal cord) led us to prioritize this candidate gene for functional analysis in mouse models.

### Anatomical analysis shows that *NCX3* is expressed in projection neurons and excitatory and inhibitory interneurons

The association between the *NCX3* locus and pain wind-up led us to investigate the expression of *NCX3* in the mouse dorsal root ganglion (DRG) and the spinal DH. Due to previous studies having identified NCX3 expression in the DRG, we first wanted to confirm this using *in situ* hybridization (ISH) and immunohistochemistry (IHC). We found *NCX3* ISH signal in all DRG neuron subpopulations tested (Figures S2F–S2L), with the strongest expression observed in large myelinated, NF200-positive DRG neurons. The TH, IB4, and CGRP DRG neuron subpopulations were more weakly stained (Figure S2I).

**Figure 2:**
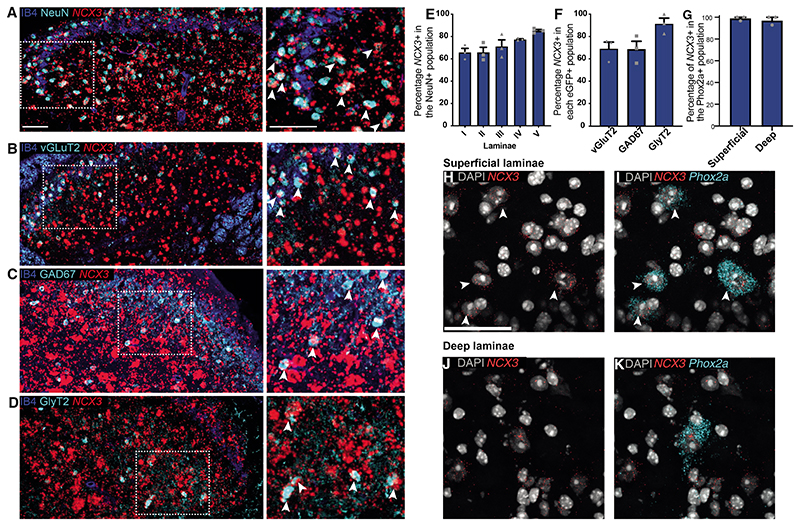
*NCX3* mRNA is strongly expressed in interneurons and projection neurons in the spinal DH (A) Representative composite image of *NCX3 in situ* hybridization (ISH) combined with immunofluorescence for NeuN (cyan) and IB4 (blue). (B-D) Representative composite images of ISH combined with immunofluorescence for interneuron markers in the spinal cord (GAD67-eGFP, GlyT2-eGFP, and vGluT2-eGFP). Right panels show magnified inserts. (E) Quantification of the data from (A)–percentage of *NCX3*-positive cells in the NeuN-positive neuronal population in laminae I to V in the spinal DH (3 images per animal, n = 3 animals). (F) Quantification of the images from (B) to (D)–data were quantified as percentage of *NCX3*-positive cells in the respective eGFP-positive populations (3 images per animal, n = 3 animals). (G) Quantification of the images from (H) to (K)–percentage of *NCX3*-positive cells in the *Phox2a* population (n = 3 animals). Scale bars, 50 μm. (H–K) Example composite images showing multiplex ISH for *NCX3* and tdTomato (labeling *Phox2a*-positive projection neurons) mRNA in the spinal dorsal horn. and (I), co-localization in the superficial laminae. (J) and (K), co-localization in the deep laminae. Scale bars, 50 μm.

As detailed anatomical analysis of *NCX3* expression in the mouse spinal cord is lacking, we conducted ISH revealing that around 65% of all NeuN-positive neurons co-localized with *NCX3* in the superficial laminae of the spinal cord. This co-local-ization was even stronger in the deeper laminae where wind-up is pronounced, with around 80% of the neurons also expressing *NCX3* (Figures 2A and 2E). We next explored the types of neurons that co-localized with *NCX3* mRNA. We used three eGFP reporter mouse lines labeling excitatory glutamatergic neurons (vGluT2-eGFP mice, Gong et al., 2003; inhibitory GABAergic neurons [Gad67-EGFP mice], Tamamaki et al., 2003; or inhibitory glyci-nergic neurons [GlyT2-eGFP mice], Zeilhofer et al., 2005). We observed *NCX3* expression in both excitatory and inhibitory DH neurons with staining identified in 91.4% ± 5% of the GlyT2 population, 68.8% ±7% of the Gad67 population, and 69.1% ± 6% of the vGlut2 population (Figures 2B–2D and 2F).

Next, we explored the expression of *NCX3* in spinal projection neurons marked with *Phox2a* or *Lypd1* (Häring et al., 2018; Roome et al., 2020). Multiplex ISH revealed co-localization in both superficial and deep laminae, suggesting the presence of *NCX3* in DH projection neurons (Figures 2G–2K and S2A–S2D). As *Phox2a* is highly expressed and marks projection neurons during development, we used a Phox2a^Cre^/Rosa26LSL-tdTo-mato mouse line and ISH for tdTomato. In this mouse, >95% of all Phox2a-tdTomato neurons are projection neurons, as confirmed by Ctb injection into the parabrachial nucleus. Additionally, up to 70% of all Ctb-labeled neurons are Phox2a positive (Alsulaiman et al., 2021). Quantification showed that *NCX3* was present in 98.9% ± 1.1% of all superficial Phox2a-positive projection neurons and in 97.6% ± 2.3% of all deep projection neurons (Figure 2G).

To summarize, in the DRG, *NCX3* was highly expressed in low-threshold mechanoreceptor afferents and at a lower level in nociceptive afferents. In the spinal cord, *NCX3* was expressed both in excitatory and inhibitory DH interneurons as well as in projection neurons of the anterolateral system, which are linked to nociceptive transmission.

### Deletion of *NCX3* is associated with normal acute-pain-related behavior but hypersensitivity to the second phase of the formalin test and chronic constriction injury

**Figure 3:**
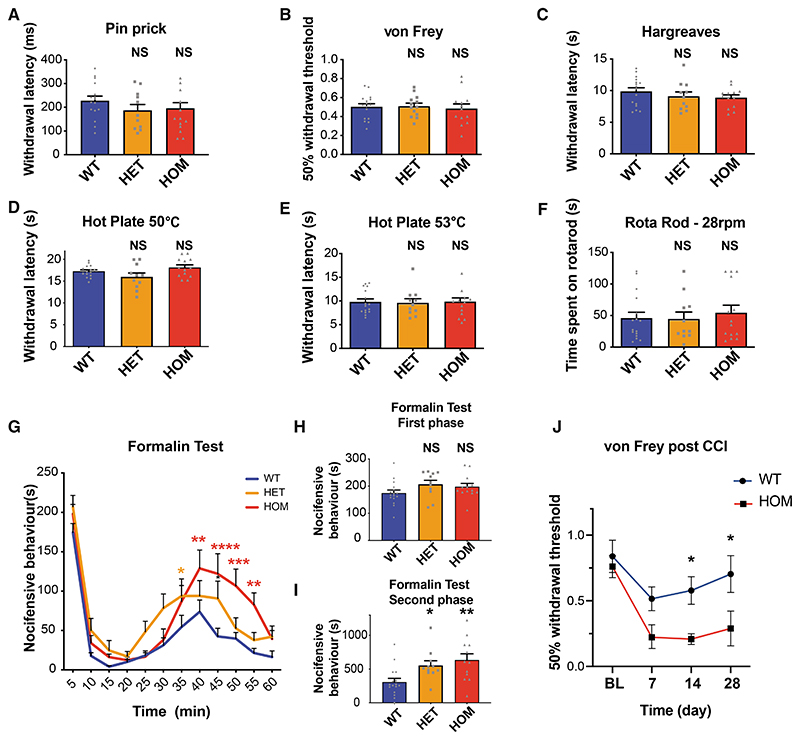
Mice lacking *NCX3* show normal motor and acute pain behavior but hypersensitivity in the second phase of the formalin test (A–E) Behavioral response to acute sensory stimuli. (F) Rota-rod assessment of sensorimotor function in WT, *NCX3*^HET^, and *NCX3^HOM^* mice. (G) Formalin test. (H) Mean results from the first phase of the formalin test (first 5 min). (I) Results from the second phase of the formalin test (20–60 min). Data from behavioral tests are mean ± SEM, WT n = 16, *NCX3^HET^* n = 11, and *NCX3^HOM^* n = 13. (J) von Frey at baseline (BL) and days 7, 14, and 28 post-chronic constriction injury (CCI). Data are mean ± SEM, WT n = 8, and *NCX3*^HOM^ n = 8. Significance shows comparison with WT (NS p > 0.05, *p< 0.05, ** p < 0.01, *** p < 0.001, **** p < 0.0001). Data analysis: one-way ANOVA, Dunnett’s multiple comparisons test for all bar charts; two-way ANOVA, Dunnett’s multiple comparisons test for formalin scatterplot.

To investigate whether NCX3 may modulate pain behavior, we performed behavioral experiments in wild-type (WT) versus NCX3 heterozygous (*NCX3*^HET^) and NCX3 homozygous (*NCX3*^HOM^) mutant mouse lines lacking functional NCX3 (Sokolow et al., 2004). We did not observe significant differences in the open field, rota-rod, or beam walk tests of motor behavior/ coordination (Figures 3F and S3A–S3C). Mechanical withdrawal threshold to von Frey hair application and latency of paw withdrawal from noxious pin prick also did not significantly differ between genotypes. Similarly, reflex withdrawal in response to a hot plate or radiant heat source was comparable between genotypes (Figures 3A–3E). Additionally, we did not see a significant change in nocifensive behavior in response to intraplantar injection of the algogen capsaicin (which activates TRPV1; Figures S3D and S3E; Caterina et al., 1999).

Next, we evaluated the first (acute) and second (sensitized) phases of the formalin response, which are believed to represent peripheral inflammation and central sensitization mechanisms, respectively (Tjølsen et al., 1992; Yoon et al., 2005; Haley and Dickenson, 2016). The first phase showed no significant differences between genotypes, consistent with our results from acute mechanical and thermal stimulation. However, in the second phase, both *NCX3*^HET^ and *NCX3^HOM^* mice displayed significantly enhanced nocifensive behavior versus WT mice; this difference was gene dosage-dependent and most marked in the *NCX3*^HOM^ group (Figures 3G–3I). We also studied the chronic constriction injury (CCI) of the sciatic nerve as a model of neuropathic pain and observed that *NCX3*^HOM^ mice demonstrated enhanced mechanical hypersensitivity versus WT mice (Figure 3J).

### Absence of *NCX3* enhances Ca^2+^ responses within DH neurons

NCX3 is a high-threshold Na^+^/Ca^2+^ exchanger (Blaustein and Lederer, 1999; Dipolo and Beauge, 2006; Scheff et al., 2014), and given its expression in DRG neurons (which ultimately project into the DH of the spinal cord), we first examined Ca^2+^ dynamics in cultured DRG neurons (Figures S4 and S5) in response to K^+^-mediated depolarization. We saw no significant difference for any of the properties tested (baseline [BL] and peak Ca^2+^, area under the curve [AUC], and exponential decay constant [tau]) when examined as a group or when split into small versus medium/large DRG neurons (Figure S5).

To investigate the effects of NCX3 on DRG soma excitability more directly, we performed whole-cell patch-clamp electrophysiology and revealed complex changes in excitability: small neurons from *NCX3*^HOM^ mice exhibited a more depolarized resting membrane potential (Table S5), and small and large (but not medium) neurons demonstrated a lower threshold for action potential generation (reduction in rheobase) (Figures S6A, S6E, and S6G). When neurons were challenged with prolonged current injections (500 ms, D50 pA up to 1 nA), fewer small-sized *NCX3^HOM^* neurons repetitively fired APs compared with WT. Of those, small-sized *NCX3*^HOM^ DRG neurons that do repetitively fire more APs are evoked at current injections ranging from 0 to 300 pA (~2–3 × rheobase), but significantly fewer APs at current injections that were greater than this (Figures S6B–S6D). Medium and large DRG neurons from NXC3^HOM^ mice fired more APs to prolonged current injections compared with WT mice (Figures S6F and S6H). Across all cell sizes, we observed progressive membrane depolarization following prolonged current injections in *NCX3^HOM^* neurons (most strikingly observed in small cells) compared with WT neurons (Figures S6F–S6L). The eventual reduction in repetitive AP firing following current injections was likely due to this progressive membrane depolarization resulting in a depolarizing block.

We also examined axonal conduction properties using compound action potential (CAP) recordings of mouse saphenous nerves. We observed no differences in the conduction properties between WT or *NCX3^HOM^* nerve properties (Figures S6M–S6R). There was no significant difference in the amplitude of A- or C-fiber CAP. Low-frequency (0.25 Hz) repetitive (x16) electrical stimulation did not cause activity-dependent slowing (ADS) of C-fiber CAPs in either group (a low-frequency repetitive stimulus used in later experiments) (Figure S6P). However, a higher frequency (2 Hz) repetitive (×16) electrical stimulus led to a small amount of ADS that was increased when the 2-Hz stimulus was continued for 2 min (×240) (Figures S6Q and S6R). The C-CAP ADS was not significantly different between WT and *NCX3*^HOM^ mice.

To summarize, the absence of *NCX3* in DRG neurons did not significantly alter Ca^2+^ dynamics but had complex effects on DRG soma excitability. Small cells (i.e., mostly nociceptors) showed excessive membrane depolarizing block at suprathreshold stimulation. Importantly, we show that conduction in sensory axons remains normal.

**Figure 4:**
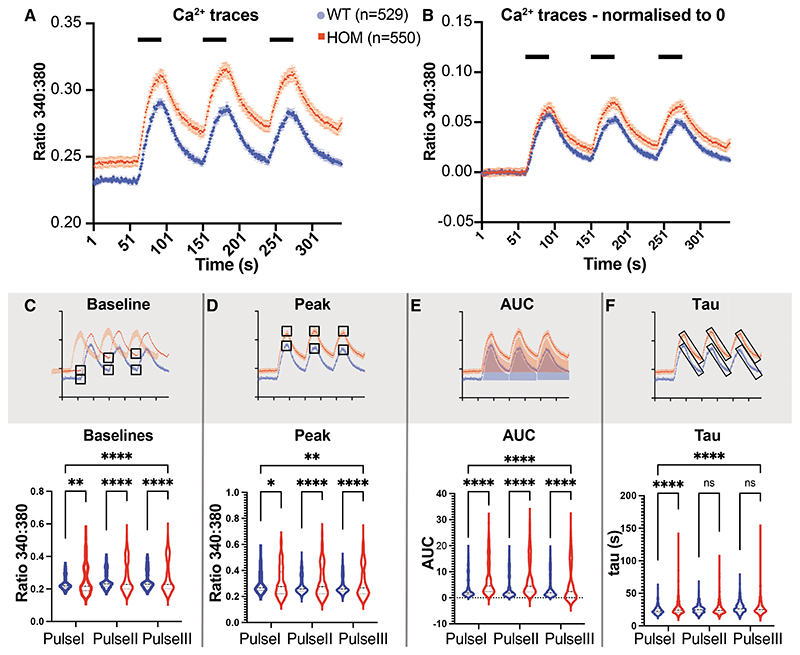
Altered Ca^2+^ dynamics in cultured *NCX3^HOM^* DH neurons (A) Mean Ca^2+^ traces during 3 K^+^ pulses (mean ± SEM). (B) Mean Ca^2+^ traces after baseline normalization. (C–F) Calcium imaging parameters–baselines, peaks, AUC, exponential decay constant (tau) shown as violin plots. One-way ANOVA (C)–(F) and Tukey multiple comparisons test. Significance shows comparison with WT *p < 0.05, **p < 0.01, **** p < 0.0001). n = 529 WT and 550 *NCX3*^HOM^ DH neurons, from WT n = 5 and *NCX3*^HOM^ n = 5 mice.

These observations in the DRG could not fully explain temporal summation of pain following repetitive noxious stimulation or our behavioral results, so we then focused on the DH. The NCX family is thought to be one of the main Ca^2+^ clearing mechanisms in the DH (Hagenston and Simonetti, 2014). We therefore examined the impact of *NCX3* on Ca^2+^ dynamics in DH neurons performing *in vitro* ratiometric Ca^2+^ imaging in DH cultures. Notably, these cultures include DH neurons and glia such as astrocytes but lack primary afferent input, enabling the investigation of the intrinsic properties of DH neurons (Albuquerque et al., 2009). We measured the response to three consecutive stimulations induced by K^+^-mediated depolarization (Figure 4A). In *NCX3^HOM^* neurons both the BL Ca^2+^ and peak Ca^2+^ responses were increased in comparison with WT (Figure 4A). Even after normalizing the BLs to zero (to account for BL changes), the difference in peaks persisted, especially at pulses II and III (Figure 4B). The BLs, peaks, and AUCs of the Ca^2+^ responses were all significantly increased in *NCX3*^HOM^ compared with WT for all three pulses (Figures 4C–4E). We also noted an impact of *NCX3* on the decay kinetics, which was quantified by assessment of tau and significantly increased in the absence of *NCX3* but only after the first stimulation (Figure 4F). This is also visualized in Figure 4B—after normalization to account for initial BL differences, the *NCX3*^HOM^ mice demonstrate increased plateaus after each peak. This slowing of decay kinetics could contribute to the phenomenon of summation during repetitive stimulation.

**Figure 5:**
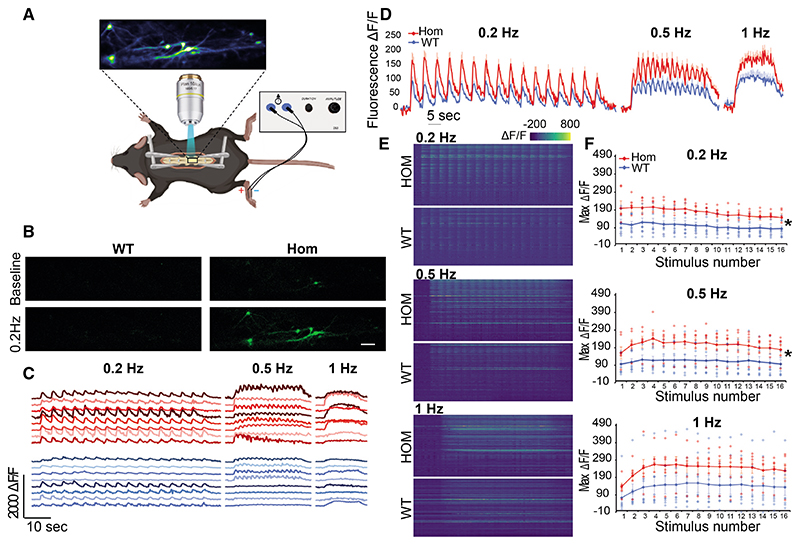
*In vivo* Ca^2+^ imaging of WT versus *NCX3*^HOM^ lamina I projection neurons (A) Setup: a laminectomy in an anesthetized mouse (WT and *NCX3*^HOM^) was used to expose the lumbar spinal cord. Standard single-photon microscopy was then applied to visualize calcium transients in lamina I projection neurons labeled with GCaMP6s. Electrical stimuli were applied across the plantar surface of the paw while calcium responses in neurons were visualized. (B) Sample images of lamina I projection neurons labeled with GCaMP6s at baseline and during electrical stimulation. Scale bars, 100 μm. (C) Sample fluorescence traces of neurons in *NCX3*^HOM^ (red) and WT (blue) mice during 0.2-, 0.5-, and 1-Hz electrical stimulation. (D) Summary trace of all responding cells (see definition of response in image processing and statistical analysis section). Data displayed as mean of WT n = 94, *NCX3*^HOM^ n = 98 + SEM. (E) Heatmap of all recorded cells during electrical stimulation. WT n = 111 cells, *NCX3^HOM^* n = 118 cells. Average of the maximal fluorescence intensity of each animal (dots). Average of WT n = 8, *NCX3*^HOM^ n = 6 displayed as line ± SEM. *p < 0.05.

In order to examine DH Ca^2+^ dynamics *in vivo*, we performed calcium imaging of lamina I projection neurons in *NCX3*^HOM^ and WT mice. An adeno-associated virus (AAV) expressing the genetically encoded calcium indicator GCaMP6s was injected into the lateral parabrachial nucleus to transduce contralateral lamina I projection neurons. These neurons were visualized in the exposed spinal cord of an anesthetized mouse, using standard single-photon microscopy (Figures 5A and 5B). Electrical stimuli applied across the mouse paw, sufficient to activate C-fiber nociceptors at different frequencies, resulted in increased GCaMP6 fluorescent signal, indicating increased intracellular Ca^2+^ in lamina I projection neurons (Figures 5B and 5C). This effect was obvious in the peak Ca^2+^ fluorescence at the first and each subsequent stimulation (Figure 5D). Indeed, the average fluorescent signal was markedly enhanced in *NCX3*^HOM^ mice, compared with WT mice (Figure 5F), at 0.2 and 0.5 Hz but not significantly at 1-Hz stimulation.

*NCX3*-deficient DH neurons demonstrated raised intracellular calcium and slowed calcium clearing following stimulation. This led us to hypothesize that the increased intracellular calcium could cause an increased response of DH neurons to noxious stimuli, especially during repetitive stimulation.

### Loss of *NCX3* leads to enhanced DH wind-up and increased nociceptive spinal-circuit excitability

**Figure 6:**
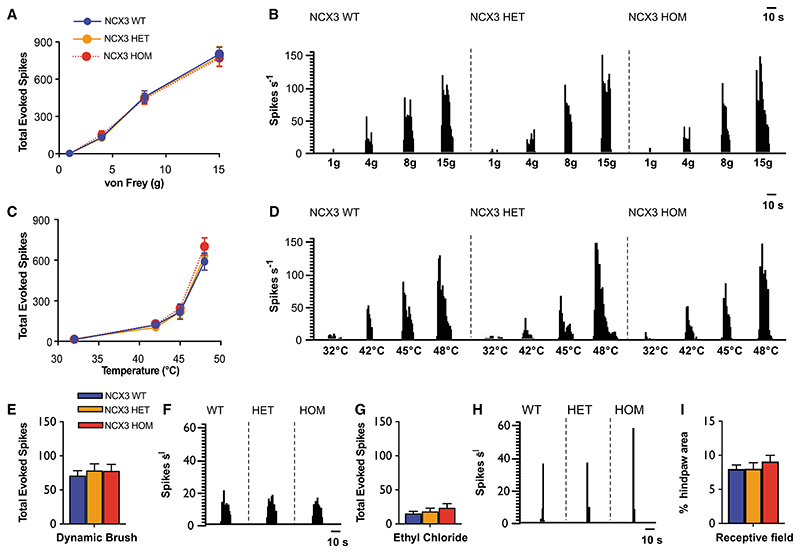
*In vivo* electrophysiology suggests normal sensory coding of deep dorsal horn neurons in WT, NCX3^HET^, and NCX3^HOM^ mice (A–H) (A) and (C), comparable evoked neuronal responses to punctate mechanical (A) and heat stimulation (C). (B) and (D), histogram traces depicting representative single unit responses. (E) and (F), evoked neuronal responses to dynamic brush and histogram traces of single unit responses. (G) and (H), evoked neuronal responses to noxious evaporative cooling and histogram traces of single unit responses. Receptive field size to a noxious punctate mechanical stimulus. Data represent mean ± SEM. WT n = 21, *NCX3*^HET^ n = 20, *NCX3*^HOM^ n = 20. No significant changes found.

**Figure 7:**
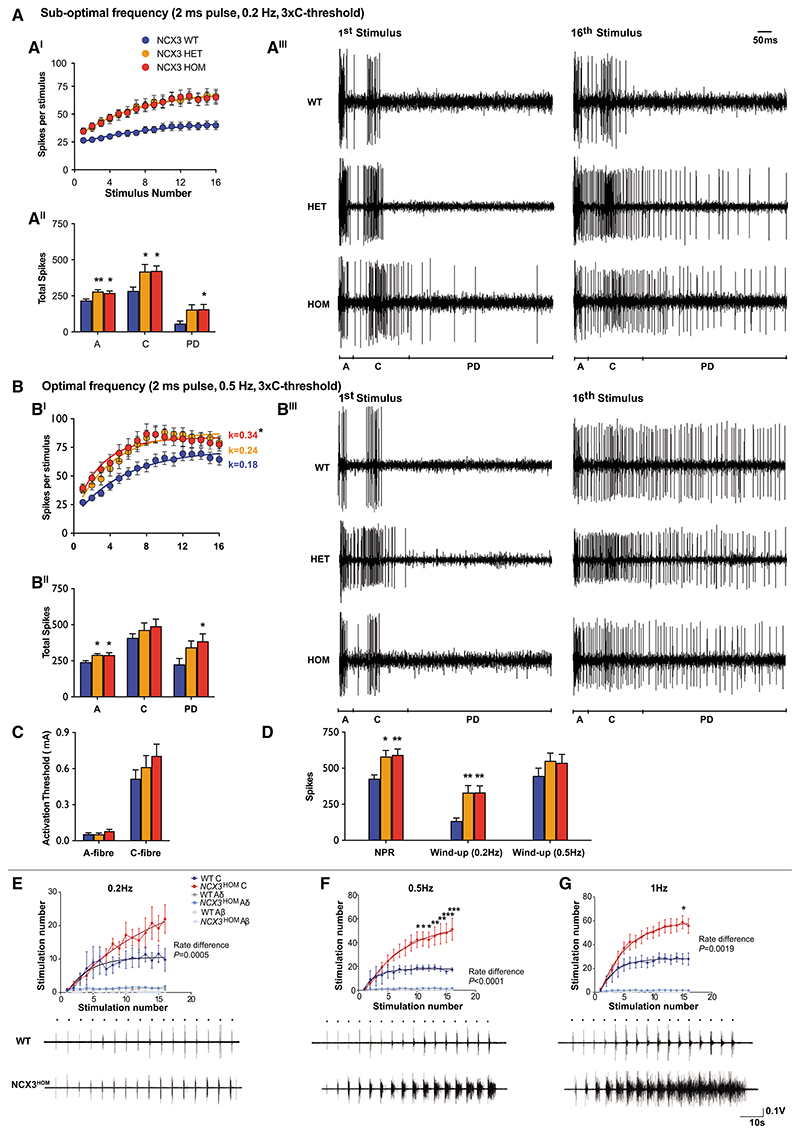
Increased wind-up and flexion reflex motoneuron responses in *NCX3* mutant mice versus WT controls (A) Wind-up of deep DH neurons (0.2 Hz) expressed as mean number of spikes per stimulus number. (A^I^) Total spikes evoked separated according to latency: A: 0–50 ms; C: 50–250 ms, PD (post-discharge) > 250 ms (A^II^). Representative spike traces (A^III^). (B) Wind-up of deep DH neurons (0.5 Hz) expressed as mean number of spikes per stimulus number. Curve analysis was performed, and rate constants are displayed. Statistics show comparison with WT. (B^I^) Total spikes evoked separated according to latency (B^II^), representative spike traces (B^III^). (C) Electrical thresholds for activation of A- and C-fibers. (D) Non-potentiated response (NPR) and wind-up following 0.2- and 0.5-Hz stimulation. Data represent mean ± SEM. Reflex responses and example traces at 0.2, 0.5, and 1 Hz. Note the increased response was observed at C-fiber (i.e., nociceptor) latency in the *NCX3* mutant mice, and there was no change at Aδ or Aβ. Significance was assessed with two-way ANOVA (A) or one-way ANOVA (A)–(D), Bonferroni multiple comparisons test. *p < 0.05, **p < 0.01. WT n = 21, *NCX3^HET^* n = 20, *NCX3*^HOM^ n = 20. Two-way ANOVA and Sidak’s multiple comparisons test were applied in (E)—WT n = 5, *NCX3*^HOM^ n = 5 *p < 0.05, **p < 0.01, ***p < 0.00. Rate constant was calculated using non-linear regression analysis (p = 0.0005).

To directly measure the electrophysiological response of DH neurons to sensory stimuli, we performed *in vivo* electrophysiology and recorded from wide dynamic range (WDR) neurons in lamina V/VI (recording depths were: WT 547 ±22 μm; *NCX3*^HET^ 593 ±15 μm; *NCX3*^HOM^ 598 ±19 μm). A range of innocuous and noxious natural stimuli were applied to the receptive field. There were no genotype differences in the evoked neuronal responses to punctate mechanical and heat stimuli (Figures 6A–6D) across a range of stimulus intensities. In addition, there were no genotype differences in the neuronal responses to dynamic brushing and noxious evaporative cooling of the receptive field (Figures 6E–6H). Receptive fields were mapped with a 15 g von Frey and were also comparable between genotypes (Figure 6I). Wind-up of WDR neurons was calculated following a train of electrical stimuli delivered transcutaneously. We found no difference in the current thresholds for activation of A- and C-fibers (Figure 7C). Compared with WT mice, however, the mutants had an increased non-potentiated response (NPR), (WT 428.2 ± 26 spikes, *NCX3*^HET^ 580 ± 42 spikes, *NCX3*^HOM^ 592.4 ± 40.5 spikes; Figure 7D). In response to a sub-optimal stimulation frequency (0.2 Hz), neurons from WT mice exhibited minimal increases in activity following repetitive stimulation (Figures 7A–7A^III^ and 7D), whereas neurons from *NCX3*^HET^ and *NCX3*^HOM^ mice exhibited higher levels of wind-up. This effect is visualized in Figure 7A^II^, where the increased number of spikes at each consecutive stimulus is obvious for the *NCX3* mutants and missing in the WT controls. As a result, the wind-up in WT mice was 135.2 ± 20 spikes, whereas for the mutants it was almost tripled (*NCX3*^HET^ 330.7 ± 48 and *NCX3*^HOM^ 332.3 ± 45; Figure 7D). These excess spikes were attributed to elevated neuronal activity in the A- and C-fibers and post-discharge (PD) latency ranges (Figure 7A^II^). By contrast, at an optimal stimulation frequency (0.5 Hz), the degree of wind-up did not significantly differ between genotypes (Figures 7B^I^-B^II^ and 7D), and we recorded an excess of 447.6 ± 53 spikes for WT, 552.2 ± 52 for *NCX3*^HET^, and 537.8 ± 57.1 for *NCX3*^HOM^ neurons (Figure 7D). We did, however, observe increased numbers of neuronal events in the A-fibers and PD latencies (Figure 7B^II^). Accelerating and plateau phases were observed at this stimulation frequency (Figure 7B^I^), in contrast to the slow progressive increase in activity seen at 0.2-Hz stimulation. Although the overall degree of wind-up was not different following 0.5-Hz stimulation, non-linear regression revealed a significantly increased rate constant for *NCX3*^HOM^ (0.336) versus WT (0.177), indicating a faster rate of wind-up during the acceleration phase (Figure 7B^I^).

We next assessed the “output” of the nociceptive reflex after repetitive stimulation *in vivo*. Afferent input from nociceptors results in the activation of motoneurons (and limb withdrawal from a noxious stimulus) via a polysynaptic spinal interneuronal network, termed the flexion reflex. This provides a means to assess spinal excitability at the circuit level (Woolf, 1983; Cook et al., 1986). Using electromyography, we measured theflexion reflex following repetitive cutaneous electrical stimulation sufficient to activate C-fibers. We observed an increased number of motoneuron spikes at C-fiber latency in *NCX3*^HOM^ mice versus WT. This effect was most pronounced during 0.5-Hz stimulation via pin electrodes in the skin of the hindpaw, but also present at 0.2 and 1 Hz (Figures 7E and S7). Additionally, the rate of motoneuron spike increase at the C-fiber latency was significantly higher in the *NCX3*^HOM^ mice at all stimulation frequencies (0.2, 0.5, and 1 Hz; Figure 7E). The flexion reflex was not enhanced at Aß latency, arguing for selective effects on nociceptive circuits and against a non-nociceptive increase in motoneuron excitability.

### *NCX3* overexpression in the DH reduced the second phase of formalin-evoked pain

**Figure 8:**
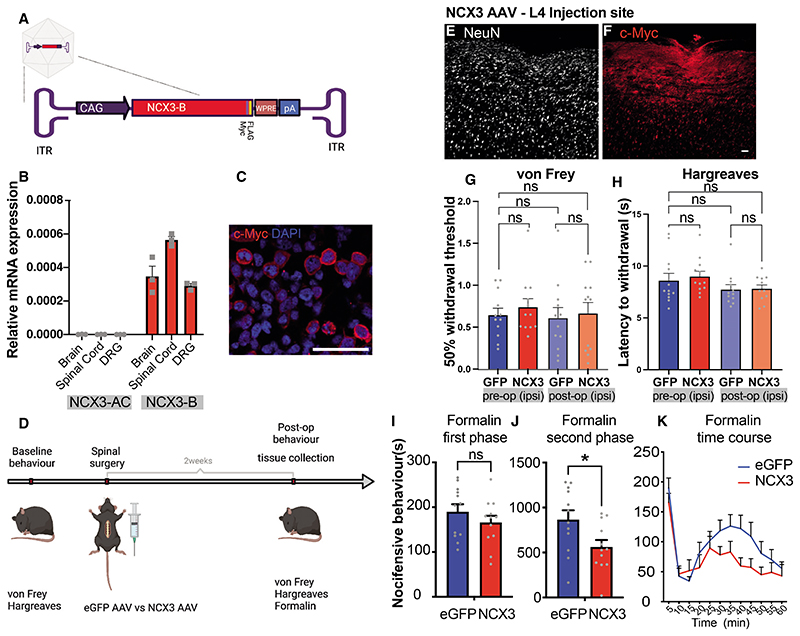
Spinal administration of viral *NCX3* reduces the second phase of the formalin response (A) Design of AAV, expressing the *NCX3-B* gene, tagged with c-Myc. (B) Splice variant *NCX3-B*, and not *NCX3*-AC, is neuronally expressed. (C) Viral transduction of HEK cells leads to membranous expression of *NCX3*-B. (D) Experimental timeline. (E and F) Expression of *NCX3*-B (c-Myc) in spinal cord sagittal sections. (G and H) Behavioral response to mechanical and thermal acute stimulation. (I–K) (I and J) Nocifensive behavior to first and second phase of the formalin test. (K) Formalin response over time. Data are mean ± SEM, eGFP n = 12, NCX3 n = 12. NS p > 0.05, *p < 0.05. Unpaired Student’s t test. Scale bars, 50 μm.

To determine whether enhanced *NCX3* expression can reduce spinal sensitization, we designed an AAV carrying the *NCX3* splice variant B (brain) tagged with c-Myc. *NCX3*-B was used because, unlike the alternative variant *NCX3-AC* (muscular) (Michel et al., 2014), it is the predominant isoform expressed in the spinal cord (Figures 8A and 8B). Viral transduction of human embryonic kidney (HEK) cells demonstrated the successful production and trafficking of *NCX3* to the membrane (Figure 8C). In addition, we observed a significant decrease in the Ca^2+^ response post ionomycin treatment of HEK cells overexpressing *NCX3* versus controls (Figures S8A–S8F).

The impact of spinal DH injections of *NCX3* or control (eGFP-expressing) AAV on pain behavior was then assessed in C57BL/6 mice. *NCX3* was effectively expressed by DH neurons (and not by DRG neurons) following intraspinal injection (Figures 8E, 8F, and S8J–S8N). There was no significant difference in acute withdrawal thresholds to von Frey hair or radiant heat (Hargreaves test) (Figures 8G and 8H). There was, however, a significant reduction of nocifensive response in the second phase of the formalin test (Figures 8I–8K), suggesting reduced central sensitization within the spinal cord. This reduction showed a significant negative correlation with the degree of *NCX3* expression at the level of L4 of the spinal cord (Figures 8G–8I).

## Discussion

Undertaking an experimental pain model in humans, we have found a novel genome-wide association between variants in the *SLC8A3* locus (encoding NCX3) and WUR (a measure of temporal summation of pain). NCX3 was shown to be highly expressed by DH neurons, and even though genetic ablation of *NCX3* in the mouse did not alter acute pain thresholds, it did result in enhanced second phase responses of the formalin test and increased mechanical pain-related hypersensitivity in the CCI model of neuropathic pain. NCX3 was shown to regulate Ca^2+^ dynamics in DH neurons and in the absence of *NCX3* electrophysiological wind-up was enhanced and could be achieved at a low frequency, providing a molecular and electrophysiological correlate of the behavioral observations. Viral overexpression of NCX3 in the DH led to reduced pain behavior in the second phase of formalin, and so enhanced NCX3 expression may have therapeutic potential.

Human experimental pain models provide the opportunity to study pain perception using stimuli that are carefully controlled in terms of modality, location, intensity, and temporal profile. There have been several candidate gene studies but relatively few GWAS studies using such experimental pain models, likely reflecting the challenges of capturing this complex phenotype in sufficient participant numbers. One study evaluated association of SNPs (revealed by exome sequencing) in a subset of twins with extremes of heat pain sensitivity; although the threshold for genome-wide significance was not reached, pathway analysis revealed significant enrichment for variants in genes of the angiotensin pathway (Williams et al., 2012). We used a validated protocol for WUR as a means to study temporal pain summation in young healthy participants to mitigate confounders such as chronic disease and aging (Schmid et al., 2019). A punctate mechanical stimulus was used (a von Frey hair), and so the pain intensity ratings were expectedly low in our healthy participants. However, repetitive stimulation showed highly significant temporal summation, and the sensory phenotype did not serve as a clinical outcome but rather identified biologically relevant gene associations. Indeed, we found three genome regions that exceeded the genome-wide significance threshold for association with WUR in the sample studied here. The evidence is most compelling for 14q24.2, where a number of associated SNPs overlap the *NCX3* gene, and this was the focus of our subsequent functional studies in the mouse. The single SNP showing association on 1q21.3 is intergenic; however, it is in proximity to (and an eQTL for) the gene S100A16, again highlighting calcium homeostasis as this gene encodes an EF-hand containing calcium-binding protein (Sturchler et al., 2006). *NCX3* is highly expressed in the CNS, and consistent with this, recent GWAS have associated variants in *NCX3* with anhedonia and insomnia (Lane et al., 2019; Ward et al., 2019) with suggestive association having been also found for bipolar disorder (Izard and Vandenbergh, 1982; Stahl et al., 2019). Since SNPs in 14q24.2 associated with WUR are non-coding, their phenotypic impact is most likely mediated through regulation of gene expression in the region, possibly of *NCX3*. The SNPs showing significant association in the Colombian sample overlap introns 2–6 of *NCX3*, a region that includes several enhancer-like signatures in the neural progenitor cells analyzed by the ENCODE consortium (The ENCODE Project Consortium et al., 2020). Furthermore, GTex data indicate that several SNPs in the *NCX3* region represent eQTLs impacting on the expression of *NCX3* in the brain (The GTEx Consortium, 2020). Although no such evidence is available for the SNPs associated here with WUR, this can be explained by these SNPs being monomorphic in Europeans (Table S1) and the current paucity of gene expression data in African ancestry populations. It is very relevant to note here that most of the current GWAS participants are of European ethnicity (Gurdasani et al., 2019), and therefore the studies are missing out on the huge genetic diversity in the rest of the world. Studies of ethnically diverse populations are crucial for a broader understanding of the biological basis of many traits; e.g., by working with the admixed Latin American populations, the CANDELA consortium was able to discover the contributions to multiple aspects of human physical variation by SNPs that are unique to Native American and East Asian ethnicities (Adhikari et al., 2016, 2019).

We used mouse models to validate NCX3 as a pain gene and to determine the impact of NCX3 levels on neuronal function in nociceptive circuits. In terms of localization, NCX1, NCX2, and NCX3 isoforms have all been reported as being expressed in DRG neurons, although there are differing reports as to relative expression within DRG neuron subsets. Using immunostaining, Persson et al. reported that NCX2 was the predominant NCX expressed within small diameter DRG neurons (and that *NCX3* showed low levels of expression in all DRG subtypes) (Persson et al., 2010). Using single-cell RT PCR in nociceptors, functional assays, and pharmacological inhibitors, Scheff et al. suggested that all 3 NCX isoforms are expressed in DRG and that NCX2 and NCX3 are particularly relevant for NCX activity in IB4-binding nociceptors (Scheff et al., 2014). Using ISH, we found that *NCX3* is expressed in DRG neurons particularly in large diameter myelinated afferents most of which are low-threshold mechanoreceptors. It is also expressed in nociceptors (both the CGRP-expressing and IB4-binding populations), albeit at lower levels. This pattern is consistent with the recently published single-cell RNA-seq dataset (Zeisel et al., 2018; Figure S2E).

We found no effect of *NCX3* ablation on Ca^2+^ dynamics in response to stimulation of DRG neurons. We did, however, discover some complex effects on excitability when undertaking patch-clamp electrophysiology at the soma: small-sized *NCX3*^HOM^ DRG neurons demonstrated a depolarized resting membrane potential, and small and large DRG neurons exhibited a lower rheobase. In regard to repetitive firing, fewer *NCX3^HOM^* small cells repetitively fire. In addition, those *NCX3*^HOM^ neurons that do repetitively fire, fire more at lower current injections and less at higher current injections. This latter effect was likely a consequence of progressive depolarization on current injection and hence a depolarizing block. The probable cause for this is that NCX is electrogenic and voltage sensitive with a reversal potential around –40 mV (Török, 2007). As membrane potential increases more than –40 mV, *NCX3* would switch from forward to reverse mode (extrusion of three Na^+^ ions and influx of one Ca^2+^), and this would oppose further membrane depolarization. In the absence of *NCX3*, this compensatory effect on membrane potential is lost. However, this mechanism may be more important for soma excitability as we found that axonal conduction properties were normal in *NCX3*^HOM^ mice including following repetitive stimulation.

We then focused on nociceptive processing in DH neurons that is established as a key locus for nociceptive wind-up within the somatosensory nervous system. *NCX3* was found to be expressed in excitatory and inhibitory interneurons as well as projection neurons of the mouse DH, again consistent with published single-cell RNA-seq datasets from spinal cord (Häring et al., 2018; Zeisel et al., 2018; Figures 2 and S2E). In particular, there were the highest levels in the deep DH where neurons exhibiting wind-up are most pronounced (Dickenson and Sullivan, 1987). We found no effect of *NCX3* ablation in mice on reflex withdrawal to acute noxious thermal or mechanical stimuli. Tests of motor function in the form of rota-rod, beam walk, and open field were also normal (Sokolow et al. [2004] previously reported a deficit in rota-rod performance; however, this involved a much more challenging endurance protocol). Subcutaneous injection of formalin in the paw is a more tonic, noxious stimulus with two phases of nocifensive pain behavior—the first phase is thought to be an acute response to the activation of nociceptive afferents (particularly via the transducer TRPA1 [McNamara et al., 2007]) but a broad range of afferents contribute (Shields et al., 2010). We found that this first phase was not dependent on *NCX3* expression, whereas the second phase—believed to be due to a combination of peripheral inflammation and central sensitization as a consequence of the afferent barrage (Haley and Dickenson, 2016)—was increased in *NCX3*^HET^ and *NCX3*^HOM^ mice (versus WT), suggesting enhanced central sensitization as a consequence of reduced or absent *NCX3* expression. The fact that *NCX3*^HET^ had a behavioral phenotype is consistent with the hypothesis that human *NCX3* variants are associated with WUR as a consequence of changes in the level of *NCX3* expression.

Ca^2+^ is known to be an important determinant of the synaptic processes underlying wind-up and central sensitization in the DH (Haley and Dickenson, 2016). Repetitive activation of nociceptor synaptic inputs to DH neurons results in progressive membrane depolarization (Sivilotti et al., 1993), relief of the Mg^2+^-dependent block of the NMDA receptor (Dickenson and Sullivan, 1987), and increased intracellular Ca^2+^ (Luo et al., 2008). Action potential propagation in DH neurons increases Ca^2+^ as a consequence of the activation of voltage-gated calcium channels and release from ryanodine-sensitive intracellular stores (Harding et al., 2020). Increased intracellular Ca^2+^ also enhances trafficking and post-translational modifications (e.g., phosphorylation) of excitatory ion channels (such as Ca^2+^-permeable AMPA and NMDA receptors) resulting in feedforward amplification of synaptic membrane depolarization. This then leads to more longterm changes in gene expression (Fang et al., 2002; Ji et al., 2002, 2009; Geranton et al., 2007), resulting in activity-dependent plasticity (Bourinet et al., 2014; Battaglia, 2016), although evoked changes in gene expression are unlikely to be relevant to the timescale of the formalin response. More is understood regarding the mechanisms underlying Ca^2+^ influx rather than its clearance in DH neurons (Hagenston and Simonetti, 2014).

The most common mechanisms for terminating Ca^2+^ signals include Ca^2+^ extrusion by plasmalemmal systems such as NCX but also the plasma membrane calcium ATPase (PMCA) or Ca^2+^ uptake into the ER and/or mitochondria. PMCA2 expression has recently been shown to be reduced in spinal cord following inflammation or trauma, and this reduction was related to enhanced pain behavior (Mirabelli et al., 2019). Using Ca^2+^ imaging of primary DH neurons, we found that the absence of *NCX3* resulted in increased BL Ca^2+^ (pre-stimulation) as well as increased peak responses to stimulation and slowed Ca^2+^ clearance. These changes in dissociated DH neurons were independent of primary afferent input. Similar changes in Ca^2+^ dynamics had previously been noted in hippocampal neurons in mice following ablation of *NCX3* (Molinaro et al., 2011). Using *in vivo* calcium imaging, we also discovered significantly increased Ca^2+^ responses to electrical stimulation of nociceptors in lamina I projection neurons. Given these changes in Ca^2+^, which have been linked to the functional properties of DH neurons, we investigated wind-up of DH neuron responses *in vivo*. The most striking finding in NCX3^HET^ and *NCX3*^HOM^ was that although we did not find increased responses to theapplication of single noxious thermal or mechanical stimuli or alterations in receptive field size, the response to repetitive electrical stimulation was enhanced. The non-potentiated DH neuron responses were increased (consistent with the increased basal Ca^2+^ levels that we had observed), and wind-up of DH responses to repetitive stimuli could now be induced at a low frequency (0.2 HZ), which is normally sub-optimal for wind-up in WT mice. At 0.5 HZ the rate of wind-up during the acceleration phase was faster. Total action potential generation by DH neurons to the train of repetitive stimuli was increased both at 0.2 and 0.5 HZ and not only at a latency appropriate for response to A- and C-fiber input but also at a PD latency. These changes provide a neural mechanism for the enhanced pain-related behavior in the *NCX3*^HOM^ mice. Our anatomical data had revealed *NCX3* expression in both excitatory as well as inhibitory spinal interneurons, and so the overall impact of reduced NCX expression would depend on the integrated effect in these different populations. We therefore also examined the flexion reflex as a means to assess the final “output” of the polysynaptic spinal interneuronal circuits processing nociceptive inputs (Woolf, 1983; Cook et al., 1986). We found that this was also enhanced in *NCX3^HOM^* mice versus controls across a range of stimulation frequencies, suggesting that at circuit-level excitability to nociceptive inputs is enhanced. *NCX3* is expressed by motoneurons (Zeisel et al., 2018). However, there was no change in the response at a latency consistent with non-noxious Aβ sensory inputs, arguing against a non-specific effect on motoneuron excitability. *NCX3* is also expressed widely in the brain, and although we have shown clear effects on the spinal processing of nociceptive inputs, we cannot exclude that changes in higher brain centres may also contribute to the behavioral changes that we observed.

Next, we tested the therapeutic potential of *NCX3* by administering it virally in the DH of naive mice. Our aim was to test the potential of enhanced *NCX3* expression in reducing pain behavior in non-genetically modified animals. We observed that this did not affect the response to acute noxious stimulation but led to a significant reduction of pain behavior in the second phase of the formalin test.

In conclusion, our data from a human experimental pain GWAS identified association between the *NCX3* locus and temporal summation of pain. We then provided direct validation of *NCX3* as a gene that regulates wind-up and central sensitization within the spinal DH of mice. Our findings in which spinal overexpression of *NCX3* could reduce pain behavior suggest that selective activators of *NCX3* would reduce temporal summation of pain in pathological states. Selective small molecule activators of *NCX3* have not yet been developed; however, targeting of NCX isoform-specific calcium-binding domains may provide a route to such selectivity (Khananshvili, 2014).

## Star∗Methods

Detailed methods are provided in the online version of this paper and include the following:

- KEY RESOURCES TABLE
- RESOURCE AVAILABILITY
  - Lead contact
  - Materials availability
  - Data and code availability
- EXPERIMENTAL MODEL AND SUBJECT DETAILS
  - Human experiments
  - Mouse experiments
- METHOD DETAILS
  - Human experiments
  - Mouse experiments
- QUANTIFICATION AND STATISTICAL ANALYSIS
  - Human experiments
  - Mouse experiments

### Key Resources Table

**Table:** 

Reagent Or Resource	Source	Identifier
Antibodies		
Sheep anti-CGRP (1:400)	Enzo Life	Cat# BML-CA1137; RRID: AB_2050885
Rabbit anti-CGRP (1:1000)	Peninsula Laboratories	Cat# T-4032; RRID: AB_2313775
Isolectin B4 (IB4), conjugated to biotin (1:100)	Sigma-Aldrich	Cat# L2140; RRID: AB_2313663
Mouse anti-NF200 (1:250)	Sigma-Aldrich	Cat# N0142; RRID: AB_477257
Rabbit anti-NCX3 (1:1000)	Swant	Cat# 95209
Chicken anti-NeuN (1:500)	Millipore	Cat# ABN91; RRID: AB_11212808
Sheep anti-Tyrosine Hydroxylase (1:200)	Millipore	Cat# AB1542; RRID: AB_90755
Chicken anti-GFP (1:1000)	Abcam	Cat# ab13970; RRID: AB_300798
GAPDH (1:1000)	Abcam	Cat# ab181602
Donkey anti-rabbit IgG Alexa 488 (1:1000)	Thermo Fisher Scientific	Cat# A-21206; RRID: AB_2535792
Streptavidin Pacific Blue (1:500)	Thermo Fisher Scientific	Cat#S11222
Donkey anti-mouse IgG Alexa 488 (1:1000)	Thermo Fisher Scientific	Cat# A-21202; RRID: AB_141607
Goat anti-mouse IgG Pacific blue (1:500)	Thermo Fisher Scientific	Cat# P31582; RRID: AB_10374586
Donkey anti-sheep IgG Alexa 488 (1:1000)	Thermo Fisher Scientific	Cat# A-11015; RRID: AB_2534082
Goat anti-chicken Alexa 488 (1:1000)	Thermo Fisher Scientific	Cat# 11039; RRID: AB_2534096
Anti-rabbit HRP-conjugated (1:5000)	Fisher Scientific	Cat# NA934VS
Anti-mouse HRP-conjugated (1:10000)	Fisher Scientific	Cat# NA931VS
Bacterial and virus strains		
ssAAV-1/2-shortCAG-mSlc8a3_myc_FLAG-WPRE-SV40p(A) (capsid 1)	VVF - custom	This paper
ssAAV-1/2-shortCAG-EGFP-WPRE-SV40p(A) (capsid 1)	VVF - repository	Cat# v587-1
Chemicals, peptides, and recombinant proteins		
Formaldehyde solution	Sigma-Aldrich	Cat# 252549
Ionomycin	Sigma-Aldrich	Cat# I0634-1 MG
Critical commercial assays		
RNAscope 2.5 HD Reagent Kit-RED	Biotechne	Cat# 322350
RNAscope® Fluorescent Multiplex Detection Reagent kit	Biotechne	Cat# 320851
LightCycler 480 SYBR Green Master mix	Roche	Cat# 04707516001
In Fusion® Cloning kit	Takara Bio	Cat# 638909
Jet PEI DNA Transfection Reagent	Polyplus Transfection	Cat# 101-10N
Deposited data		
Raw data showing Ca2+ imaging selection protocol and decay kinetics analysis	https://github.com/Teodora-trend/calcium_imaging	https://doi.org/10.5281/zenodo.6529629
Experimental models: Cell lines		
HEK-293	N/A	N/A
Experimental models: Organisms/strains		
Slc8a3^tm1Sso^ mouse line	Sokolow et al., 2004	N/A
Gad67-EGFP mouse line	Tamamaki et al., 2003	Tissue from Professor H. Zeilhofer
GlyT2-eGFP mouse line	Zeilhofer et al., 2005	Tissue from Professor H. Zeilhofer
vGluT2-eGFP mouse line	Gong et al., 2003	Tissue from Professor H. Zeilhofer
Phox2a^Cre^ x Rosa26LSL-tdTomato		Tissue from Professor A. Todd
C57Bl/6J mouse line used for spinal injections	Charles River	Strain code 632
Oligonucleotides		
NCX3_F CAGATAAGCGACTGCTCTTC	Thermo Fisher Scientific	Sokolow et al., 2004
NCX3_R CCTGGCTTCAGAACCACAGTG	Thermo Fisher Scientific	Sokolow et al., 2004
NCX3_Neo TCGACTAGAGGATCAGCTT	Thermo Fisher Scientific	Sokolow et al., 2004
NCX3-AC_F GGGCCCCCGCATGGTGGATA	Thermo Fisher Scientific	Michel et al., 2014
NCX3-AC_R CAGCTTCCTGTCTGTCACTTCTGGA	Thermo Fisher Scientific	Michel et al., 2014
NCX3-B_F GCATATGGGGAGCTGGAGT	Thermo Fisher Scientific	Michel et al., 2014
NCX3-B_R GTTCACCAAGGGCAATGAAG.	Thermo Fisher Scientific	Michel et al., 2014
Cloning Oligo 1 (BamHI) (Insert 1) Forward GCAAAGAATTGGATCCGCCACCATG GCGTGGTTA	Thermo Fisher Scientific	This paper
Cloning Oligo 2 (Insert 1) (EcoRI) Reverse GCTTGATATCGAATTCTTAAACCTTATCGTCGTCATCCTTG	Thermo Fisher Scientific	This paper
RNAscope® Probe- Mm-Slc8a3	Biotechne	Cat# 523681
RNAscope® Probe- Mm-Lypd1	Biotechne	Cat# 318361-C2
RNAscope® Probe- Mm-tdTomato	Biotechne	Cat# 317041-C2
Recombinant DNA		
pAAV CAG-GFP	From Edward Boyden (Addgene plasmid # 37825; http://n2t.net/addgene:37825)	RRID: Addgene_37825
Slc8a3-B (Myc-DDK-tagged)	Origene	Cat# MR211189
Software and algorithms		
Plink software for genetic data analysis and GWAS	Chang et al., 2015	https://www.cog-genomics.org/plink/1.9/
R statistical software		https://cran.r-project.org/
FastMan package for R for manhattan plot visualization	Paria et al., 2022	https://github.com/kaustubhad/fastman
R package for GWAS power calculation	Schmid et al., 2019	https://github.com/kaustubhad/gwas-power
SHAPEIT2 software for genetic data phasing	O’Connell et al., 2014	https://mathgen.stats.ox.ac.uk/genetics_software/shapeit/shapeit.html
IMPUTE2 software for genotype imputation	Howie et al., 2012	https://mathgen.stats.ox.ac.uk/impute/impute_v2.html
ADMIXTURE software for genetic ancestry estimation	Alexander et al., 2009	http://dalexander.github.io/admixture/
GCTA software for gene-level association GCTA-fastBATtest	Bakshi et al., 2016	https://cnsgenomics.com/software/gcta/#Gene-basedtest
Illumina GenomeStudio software for genotype calling		https://www.illumina.com/techniques/microarrays/array-data-analysis-experimental-design/genomestudio.html
GraphPad Prism 9	GraphPad	https://www.graphpad.com/
ImageJ	National Institutes of Health	https://imagej.nih.gov/ij/
FIJI software for pixel-based color quantification	FIJI	https://imagej.net/Fiji
Avidemux	Avidemux	https://avidemux.en.softonic.com/mac
Microsoft Excel	Microsoft	https://www.microsoft.com/en-gb/
Matlab	Mathworks	https://uk.mathworks.com/products/matlab.html
BioRender		https://biorender.com
Neurolucida	MBF Bioscience	https://www.mbfbioscience.com/neurolucida-version-2020
Other		
GWAS summary statistics from the CANDELA cohort	GWAS Central	https://www.gwascentral.org/study/HGVST3308
GTEx expression data for SLC8A3	GTEx	https://www.gtexportal.org/home/gene/SLC8A3
GTEx expression data for S100A16	GTEx	https://www.gtexportal.org/home/gene/S100A16
ENCODE expression data for SLC8A3	ENCODE	https://screen.wenglab.org/geApp/?assembly=GRCh38&gene=SLC8A3

### Resource Availability

#### Lead contact

Further information and requests for resources and reagents should be directed to and will be fulfilled by the lead contact Prof. David Bennett - david.bennett@ndcn.ox.ac.uk.

#### Materials availability

Raw genotype or phenotype data of human participants cannot be made available due to restrictions imposed by the ethics approval.

### Experimental Model And Subject Details

#### Human experiments

##### GWAS - data collection and analysis

The study sample reported is a cohort characterized using quantitative sensory testing following a protocol described in detail previously (Schmid et al., 2019). Briefly, individuals aged 18-40 were recruited in Medellin, Colombia, via public noticeboards at local Universities, distribution of flyers and through the local print media. Exclusion criteria were: (i) chronic pain, (ii) any chronic medical condition (including diabetes, neurodegenerative, musculoskeletal or psychiatric disorders), (iii) use of analgesics, anti-inflammatories, opioids, antihistamines, antidepressants, or anti-epileptic medications (iv) pregnancy, or being in the menstrual phase (as self-reported), (v) dermatomal, traumatic, or infectious conditions affecting the arms (vi) current or past self-inflicted injuries (vii) a history of severe allergic reactions of any kind. Furthermore, since psychological factors can influence pain perception during experimental pain testing, participants reporting moderate to severe anxiety (≥25 on the Hamilton Anxiety Rating Scale) or severe depression (>15 on the Quick Inventory of Depressive Symptomatology (QIDS-SR16) prior to testing were also excluded (Schmid et al., 2019).

This study was approved by the bioethics committee of the Odontology Faculty at the University of Antioquia (CONCEPTO 01-2013). Some of the individuals examined here had been genotyped for the CANDELA cohort study (Adhikari et al., 2016) which was approved by University College London research ethics committee (3352/001). All participants gave written informed consent prior to participating in the study. The first cohort (discovery, n = 1061) was collected between May 2013 and Dec 2016. The second cohort (replication, n = 317) was collected between Jan 2017 and July 2019.

#### Mouse experiments

##### Histology: Mouse lines

All procedures were carried out in accordance with UK home office regulations and mouse welfare was continuously assessed throughout all procedures. The temperature and humidity of the rooms were monitored at all times and food and water were freely available. Mutant mice *Slc8a3^tm^* (Sokolow et al., 2004) were housed in IVCs, studied on a C57Bl6 strain background and both male and female animals were included in the analysis. For behavioral and electrophysiological experiments *Slc8a3*^tm1Sso, tm1Sso^ (termed *NCX3*^HOM^) were compared to wild type litter mates. Genotyping primers were as follows: NCX3 Neo - TCGACTAGAGGATCAGCTT, NCX3_F - CAGATAAGCGACTGCTCTTC, NCX3_R - CCTGGCTTCAGAACCACAGTG. Experiments are reported according to the ARRIVE guidelines. We used spinal cord tissue from three previously reported eGPF marker lines - vGluT2-eGFP (Gong et al., 2003), Gad67-eGFP (Tamamaki et al., 2003) and GlyT2-eGFP (Foster et al., 2015).

### Method Details

#### Human experiments

##### Pain sensitivity testing

Participants attended a single appointment at the quantitative sensory testing (QST) laboratory at Universidad de Antioquia, in Medellín Colombia. Smoking and drinking of coffee within one hour of testing and consumption of psycho-active substances or alcohol within eight hours of testing were discouraged. Age and self-reported gender were recorded. Participants completed the Spanish version of the Hamilton Anxiety Rating Scale (HARS with a range of 0-56, in which scores <17 indicate mild anxiety, 18-24 = mild to moderate anxiety, and 25-30 moderate to severe anxiety) and the Quick Inventory of Depressive Symptomatology Self-Report (QIDS-SR, with a range 0-27 in which 1-5 indicate no depression, 6-10 = mild, 11-15 = moderate, 16-20 = severe and 21-27 = very severe depression).

WUR was evaluated using a 255mN von Frey hair (Touch Test, North Coast, USA). Pain ratings were recorded on a numerical scale (0-100) for a single stimulus, which was then followed by a train of 10 stimuli applied at 1 Hz in the same 1 cm^2^ area as the single stimulus, and the average recorded. This procedure was repeated five times and WUR calculated as the mean pain rating for the trains of stimuli divided by the mean of the single stimulus. Participants were first familiarized with the testing midway over the contralateral ventral forearm before actual testing on the ipsilateral forearm. The testing side was randomized. We have previously shown high intra-tester reliability for wind-up ratio (ICC_3_._1_ = 0.634, (95% CI 0.113-0.880), p = 0.012) (Schmid et al., 2019).

##### DNA genotyping and filtering

DNA samples (extracted from blood or saliva) were genotyped on the Illumina HumanOmniExpress chip, including 730,525 SNPs aligned to the human genome reference sequence build GrCh37. SNPs with >5% missing data or minor-allele frequency <1%, were excluded. Due to the admixed ancestry of the study sample, there is an inflation in Hardy-Weinberg p-values (Adhikari et al., 2016). We therefore did not exclude SNPs based on Hardy-Weinberg deviation, but performed stringent quality controls at software and biological levels (see Figure S14 from Adhikari et al., 2016, and Table S2). For example, the SNP quality metrics generated from the GenCall algorithm in Illumina GenomeStudio v2.0 were used for quality control. SNPs with low GenTrain score (<0.7), low Cluster Separation score (<0.3) or high heterozygosity values (|het. excess|>0.5) were excluded (Adhikari et al., 2016). In addition, we checked for batch effects by genotyping a control sample on each plate and comparing its genotype calls across batches; the consistency rate (i.e. matched proportion of genotypes) was ≥ 0.999 in all cases after SNP-level QC. We also assessed the accuracy of our genotypes independently by comparing against genotype calls obtained on a subset of samples by high-coverage sequencing, and consistency was again ≥ 0.999. The quality control filters resulted in 673,034 SNPs being retained for further analysis. Even though we did not explicitly filter SNPs based on HWE, we show in Table S2 that all the index SNPs have non-significant HWE p-values.

#### Mouse experiments

##### Tissue preparation for histology

For immunohistochemistry and *in situ* hybridization experiments, mice were overdosed with pentobarbital and perfused transcar-dially with room temperature sterile saline, followed by 10-15 mL 4% paraformaldehyde (PFA). Once dissected, DRGs were post-fixed in 4% PFA for 2 h at room temperature, while the spinal cords were post-fixed for 24 h at 4°C. All tissue was dehydrated for cryoprotection in 30% sucrose at 4°C for 4-5 days. Optimal cutting temperature (OCT) medium (Tissue-Tek) was then used to embed the dissected tissue and allow for -80°C storage. Sections were cut on a cryostat (14 μm for DRG, 30 μm for spinal cord) and stored at -80°C. Tissue preparation for *in situ* hybridization (multiplex) was different and is specified below.

##### Immunohistochemistry and in situ hybridization

For immunohistochemistry, DRG and spinal cord sections were washed in PBS and PBS Triton-X (0.3%), before being incubated overnight at room temperature with the respective primary antibodies diluted in PBS/Triton. The primary antibodies used for immunohistochemistry: anti-NeuN (chicken, 1:500, Millipore, Cat# ABN91), anti-GFP (chicken, 1:1000, Abcam, Cat# ab13970), anti-CGRP(sheep, 1:400, Enzo Life, Cat# BML-CA1137), anti-CGRP (rabbit, 1:1000, Peninsula Laboratories, Cat# T-4032), anti-IB4 (biotin, 1:100, Sigma-Aldrich, Cat# L2140), anti-NF200 (mouse, 1:250, Sigma-Aldrich, Cat# N0142), anti-TH (sheep, 1:200, Millipore, Cat# AB1542), anti-NCX3 (rabbit, 1:1000, Swant, Cat# 95209) (key resources table). This was followed by washing in PBS/Triton and incubation for 2 hours at room temperature with secondary antibodies (Alexa Fluor, Thermo Fisher Scientific). The tissue was then washed again and cover-slipped. Immunostaining was visualized using a confocal microscope (Axio LSM 700, Zeiss) and images were acquired using the Zen black software.

For *in situ* hybridization (singleplex), we used the RNA Scope 2.5 Red chromogenic assay kit (Biotechne, 322350) and followed manufacturer instructions. Briefly, after -80°C storage, tissue was allowed to reach room temperature and was then washed with PBS. Next, tissue was pre-treated with hydrogen peroxide at room temperature (10-min pre-treatment for DRG, no pre-treatment for spinal cord) and protease at 40°C (10-min protease treatment for DRG, 15 min for spinal cord). Slides were then incubated with an mRNA probe against *NCX3* (Slc8a3) (Biotechne, cat# 523681) for 2 h at 40°C. Probe incubation was then followed by six amplification steps, with Amplification 5 lasting 30 min for DRG samples and 15 min for spinal cord tissue. All other amplification steps were performed as per manufacturer’s instructions. The development stage of the protocol involved a fast red reaction, which was also tissue-dependent: 7 min for DRGs and 10 min for spinal cords. Any modifications to the protocol were optimized to reduce background staining and improve signal. ISH was followed by immunohistochemistry, where the standard protocol described above was used. Staining was visualized with confocal microscope and images were acquired using the Zen black software.

For *in situ* hybridization (multiplex), we used RNA Scope Multiplex Kit V1 (Biotechne, cat number: 320851). This protocol was performed on fresh frozen tissue and the manufacturer’s instructions were followed. For tissue preparation, the spinal cords were dissected using hydraulic extrusion. Tissue was then quickly placed on a frozen metal plate on dry ice. After freezing, the spinal cord was transferred to a cold Eppendorf tube and stored at -80°C. A cryostat was used for tissue cutting, where the spinal cords were left to equilibrate to -20°C and were then covered with OCT. We used 12-15 μm section thickness and kept the tissue as cold as possible throughout the preparation procedure. Sections were then stored at -80°C on slides. Following tissue preparation and storage, the tissue was immediately post-fixed with 4% PFA for 15 min at 4°C. Then the slices were dehydrated and treated with probes and amplification buffers as per manufacturer’s instructions. Probes were against *Slc8a3*(NCX3), (cat# 523681), *Lypd1* (cat# 318361-C2) and TdTomato (cat# 317041-C2). TdTomato was used to mark the *Phox2a* population in *Phox2a*^Cre^;Rosa26LSL-tdTomato mice. Staining was visualized with confocal microscope and images were acquired using the Zen black software. *Phox2a* quantification was conducted manually in Neurolucida.

##### Image analysis

Analysis of the signal intensity for *in situ* hybridization studies on DRG was calculated using ImageJ. In a single image of a section of either L4 or L5 DRG, neurons were circled and the percentage coverage of red *NCX3* mRNA signal for that cell profile area was calculated. By eye each cell was subpopulation-defined using the counterstain with NF200, IB4, CGRP or TH. For each marker at least 3 sections were imaged per animal. On each image, a background reading was performed using an unstained area. The mean intensity per animal was calculated to account for any background measurement.

For spinal cord sections, mRNA positive cells were defined as those containing 3 or more red chromogen foci, colocalizing with each eGFP-marked cell (either GlyT2-eGFP, Gad67- eGFP or vGLUT2-eGFP). The total number of eGFP+ cells as well as the number of eGFP+/*NCX3*+ was counted. Double positive cells were expressed as a percentage of eGFP+ neurons.

##### Histology after spinal injections

After completion of the behavioral tests, mice were deeply anesthetized with pentobarbitone (30 mg, ip) and perfused transcardially with 4% freshly depolymerized formaldehyde in phosphate buffer. The spinal cords were then dissected out and post-fixed for 2 h in PFA. The lumbar enlargement (L3-L5 segments) was dissected and postfixed for 2 h and cut into 60 μm parasagittal sections using a vibrating blade microtome. The sections were processed for immunocytochemistry as described above. Sections were incubated in anti-GFP (chicken, 1:1000, Abcam, Cat# ab13970) or anti-Myc (rabbit, 1:1000, Abcam, Cat# ab9106) and anti-NeuN (guineapig, 1:1000, Synaptic Systems, Cat# 266004) for 3 days at 4°C and revealed with fluorescence-labelled species-specific secondary antibodies (Jackson ImmunoResearch, West Grove, PA, USA). All antibodies were diluted in phosphate-buffered saline containing 0.3% Triton-X and 5% normal donkey serum. Injection sites were assessed visually to confirm successful injection and expression of viral constructs, based on the resulting myc or eGFP signal. Additionally, the NeuN expression was assessed visually to confirm that no loss of neurons was observed within the injection zones. Based on these criteria, all 24 mice were included for further analysis (12 NCX3 and 12 eGFP). For quantitative analysis of *NCX3*-myc expression in *NCX3*-injected mice, single optical sections were scanned from the core of each injection site in each animal using a 10x objective (NA 0.3) on a Zeiss LSM900 confocal microscope in Airyscan Confocal mode (at the default zoom of 1.3x). To avoid bias when selecting the z-depth of the optical section the NeuN channel was used, with the optical section being taken at the z-level at which the NeuN signal was brightest. The imaging parameters were kept identical across all images.

To examine the correlation between the characteristics of the *NCX3* injection site and the extent to which nocifensive behavior was affected in the formalin test, we used ImageJ software. The brightest part of the L4 injection site was outlined and measurements of Integrated Density/Raw Integrated Density (the sum of the values of all pixels in the selection, providing a collective measure of the size and staining intensity) were taken. The experimenter was blind to the behavioral responses of each animal. GraphPad Prism was then used to calculate the Pearson’s correlation between each measurement and the respective behavioral response. BioRender was used to create some of the images.

##### Behavioral procedures

At the start of each set of behavioral experiments, mice, aged 8-10weeks, were acclimatized to the testing equipment for 2-3 days prior to each test. For motor and acute sensitivity tests, baseline values were obtained by averaging data from 3 experimental sessions. For behavioral experiments, we used the following two cohorts: 1^st^ cohort: 5 WT (n_female_ = 2, n_male_ = 3), 9 *NCX3*^HET^ (n_female_ = 4, n_male_ = 5) and 4*NCX3*^HOM^ (n_female_ = 3, n_male_ =1);2^nd^ cohort: 11 WT(n_female_ = 5, n_male_ = 6), 2 *NCX3*^HET^ (n_male_ = 2) and 9*NCX3*^HOM^ (n_female_ = 6, n_male_ = 3), which brings the total of mice to 16 WT, 11 *NCX3*^HET^, 13 *NCX3*^HOM^. All tests were performed in the same room, at similar times of the day, by the same experimenter, who was blind to all animal genotypes and handled the mice in a random order. Formalin experiments were performed last and therefore the animals were 20-25 weeks of age and very well acclimatized to the room and researcher. No animals were excluded from the above-mentioned cohorts.

Behavioral outcomes were obtained as described in Dawes et al. (2018). Methods for statistical analyses are outlined below.

##### Open field

For the Open field test, a black box displaying a grid system on the floor was used. Mice were acclimatized to it the day before the experiment. Before the start of the test and after every animal, the Open field box was wiped with a detergent to remove any scent clues left by previous mice. Each mouse was placed in the top left corner of the box and then allowed to explore the Open field uninterrupted for 3 min. The number of boxes entered and the rearings performed by each mouse were recorded. The rearing behavior consisted of animals standing on both hind paws in a vertical upright position. The experiment was repeated three times, on three consecutive days.

##### Rota-Rod

Motor behavior was assessed using a Rota-Rod. Prior to the experiment, mice were trained by being placed on the Rota-Rod three times the day before. Each mouse was placed on the rotating bar and the duration the mouse remained on it was measured. The cutoff time was 120 s. A constant speed of 28 rpm was used and the bar was wiped before each new mouse was placed on it.

##### Beam walk test

Proprioception was assessed using the Beam walk test as previously described. We used a 1 m long beam apparatus resting on two poles, 50 cm above a tabletop. A black box with bedding material and food was placed at the end of it to attract the mouse. A light source at the beginning of the beam was used as an aversive stimulus. The mice were placed on the beam three times the day prior to the experiment and were trained to walk to the black box. A video camera was set on a tripod to record the performance of each mouse. The experiment was repeated three times, on three separate days. The number of correct steps, as well as the number of slips and hops was recorded.

##### von Frey

Mechanical sensitivity was assessed by applying calibrated von Frey hairs (Ugo Basile) to the plantar surface of the hind paw to calculate the 50% withdrawal threshold. The von Frey hairs are nylon monofilaments which apply different force to the stimulated skin. Mouse behavior was scored using the up-down method (a statistical tool to determine the 50% withdrawal threshold through a sequential array of experiments). Using a range of stimuli, we first applied the middle weight, 0.6 g, where a negative response increased the next weight applied, and a positive one – decreased it. The resulting pattern of responses was used to select a constant k and determine the final 50% withdrawal threshold. The same experiment was performed on 3 consecutive days and the mean value for each animal was reported. For experiments involving spinal injections with *NCX3* or eGFP viruses, mice were tested once 3-5 days prior to surgery and again 2 weeks following surgery, with the experimenter blind to the virus injected.

##### Pin prick

Noxious mechanical stimulus response was assessed using the Pin prick test. Briefly, a pin was attached to a 1 g von Frey filament and then applied to the planter surface of the hind paw of each mouse. This test was done 3 times per paw, in three separate days. Videos were recorded with a Samsung mobile phone at 240 frames/s and then analyzed with the video editing program Avidemux. The latency between the pin contacting the skin and withdrawal of the hindpaw was calculated.

##### Hargreaves

Thermal sensitivity was assessed using the Hargreaves method. The mice were acclimatized to small boxes with a glass floor for 2-3 days prior to experiment. On the day of the experiment, a radiant heat source was applied to the plantar surface of the hind paw and the latency to withdrawal was recorded. This was performed 3 times for each paw and was then repeated on three consecutive days. For experiments involving spinal injections with *NCX3* or eGFP viruses, mice were tested once 3-5 days prior to surgery and again 2 weeks following surgery, with the experimenter blind to the virus injected.

##### Hot plate

Response to a supra-threshold heat stimulus was measured using the hot plate test (Ugo Basile) where each mouse was placed on a metal surface maintained at a constant temperature (50°C or 53°C). The time taken to elicit a nocifensive response (hind paw withdrawal or licking) was recorded. The cut-off time used to prevent tissue damage when no behavior is observed was 20 s.

##### Formalin

The formalin test was performed by an intraplantar injection of 20 μl formalin (5% v/v from 37% stock formaldehyde solution (Sigma, Cat#252549)). Mice were then placed in a Perspex cylinder and observed for nocifensive behavior, such as biting, licking and paw lifting. The duration of pain behavior was recorded over a 60-min period, separated into 12 5-min bins. The behavioral response is biphasic and therefore further comparisons were made by pooling data in the first (0-20 min) and second (20-60 min) phases. In Oxford, behavior was directly observed and recorded live by the experimenter. For experiments involving spinal injections with *NCX3* or eGFP viruses performed in Glasgow, mice were video-recorded 2 weeks following surgery, with the experimenter blind to the virus injected. The videos were then scored offline.

##### Chronic constriction injury (CCI)

CCI was performed based on the model described previously (Bennett and Xie, 1988). Mice were anesthetized using isoflurane inhalation and the left sciatic nerve was exposed at the high-thigh level. Two ligatures were tied loosely around the nerve, using 6-0 silk suture in an area proximal to the nerve trifurcation, at intervals of ~2 mm. The skin incision was then closed with 2-3 external stitches. All behavioral measurements were taken in awake and unrestrained mice of both sexes. One session of habituation to the testing area was conducted followed by one baseline measurement session. Von Frey experiments were performed as explained above. The experimenter was blind to all mouse genotypes. Mice that did not develop sensitivity to von Frey post-surgery (at least 25% reduction from baseline thresholds on any of the test days) were excluded. The exclusion criteria were selected prior to analysis and unblinding.

##### In vitro Ca^2+^ imaging

*DH dissection and culture*. Dorsal horn cultures were based on the protocol of Albuquerque et al. (2009). For dissection of spinal dorsal horns, we euthanized WT or *NCX3*^HOM^ mice at an age between postnatal days 6 and 12 (male and female, WT n = 5 and *NCX3^HOM^* n = 5). Spinal cords were removed and placed into ice cold Hank’s Balanced Salt Solution, HBSS (Invitrogen). Once removed, each spinal cord was placed with its dorsal side facing upward. Following a midline incision, the cord was spread open and only the outer (dorsal) one-third of each side was collected. This was followed by an enzymatic digestion for 75 min in collagenase II (4 mg/ml, Worthington) and dispase II (4.7 mg/ml, Roche) at 37°C. Then the tissue was mechanically dissociated using glass pipettes and spun down at 600xg for 6 minutes. The pelleted cells were resuspended into cell medium (Neurobasal® medium supplemented with 2% (v/v) B27 supplement, 1% (v/v) N2 supplement, 1% (v/v) GlutaMAX™ and 1.5% antibiotic/antimycotic (ThermoFisher Scientific)) and plated on 13 mm coverslips pre-coated with laminin (R&D Systems) and poly-D-lysin (BD biosciences). After allowing the cells to attach to the coverslips for 2 h at 37°C, the wells were flooded with the above-mentioned cell medium. Following this procedure, the cultures contained both neurons and glial cells. They were maintained for 2-3 weeks at 37°C, to allow time for recovery and maturation after the process of dissociation. This proved crucial for the ability of the neurons to respond to high K^+^. During the maintenance phase, the culture medium was replaced every 3-4 days.

*Dorsal root ganglia dissection and culture*. Mice aged between 4-15 weeks were euthanized using a CO_2_ chamber (Ca^2+^ imaging: male and female, WT n = 5 and *NCX3*^HOM^ n = 5, patch-clamp: male and female, WT n = 4 and *NCX3*^HOM^ n = 4). The spinal column was dissected out and cut in half through the midline. The cord was then removed to reveal the DRGs. The ganglia were taken out one by one, from all levels and placed into ice cold Hank’s Balanced Salt Solution, HBSS (Invitrogen). This was followed by an enzymatic digestion for 90min in collagenase II (4mg/ml, Worthington) and dispase II (4.7 mg/ml, Roche) at 37°C. The tissue was mechanically dissociated using glass pipettes and spun down at 500 x g for 5 minutes. The pelleted cells were then resuspended into cell medium (Neurobasal® medium supplemented with 2% (v/v) B27 supplement, 1% (v/v) GlutaMAX™ and 1% antibiotic/antimycotic (ThermoFisher Scientific)) and plated on 13 mm coverslips pre-coated with laminin (R&D Systems) and poly-D-lysin (BD biosciences). After allowing the cells to attach to the coverslips for 2 h (at 37°C), the wells were flooded with the above-mentioned cell medium, supplemented with mouse NGF (50 ng/ml, Peprotech) and GDNF (10 ng/ml, Peprotech). The prepared primary culture was then left at 37°C overnight and calcium imaging was performed the next day (1 day *in vitro* (DIV)), patch-clamp recordings were performed at 1-3 DIV.

*In vitro Ca^2+^ imaging – procedure*. In total, between 37 and 200 dorsal horn neurons from each animal, of each genotype, were imaged. Similarly, for the DRG analysis, between 21 and 67 cells were used from each animal. Coverslips were incubated for 45-90 min at 37° C with 1 μM Fura-2AM (Invitrogen) in Neurobasal medium supplemented as above. After incubation, coverslips were transferred to artificial extracellular fluid (140 mM NaCl, 5 mM KCl, 2 mM CaCl_2_, 1 mM MgCl_2_, 10 mM D-Glucose, 10 mM HEPES in distilled water). Coverslips were imaged every 1s with 4x4 binning for 340 s on a Zeiss inverted fluorescence microscope with a 10x objective, dichroic LP 409 mirror, BP 340/30 and BP 387/15 excitation and 510/90 emission filters. ZEN Blue software was used for image acquisition and selection of regions of interest (ROIs).

ECF was perfused continuously over the cells. After 60s of baseline recording, the cells were perfused with 50 mM K^+^ for 30 s (100 mM NaCl, 50 mM KCl, 2 mM CaCl_2_,1 mM MgCl_2_,10 mM D-Glucose, 10 mM HEPES). This was followed by a 60 s washout and then the K^+^ treatment/washout procedure was repeated two more times.

Data were then analyzed using GraphPad Prism (for AUC analysis) and a Matlab script (for quality control and decay kinetics analysis).

*Quality control of neurons*. Matlab was used for quality analysis. To this purpose we employed a Matlab function which allowed the selection of neurons which respond reliably to the three consecutive K^+^ pulses. Therefore, cells which showed three similar responses scored highly and were considered of high reliability. We chose the threshold of 1 and accepted only neurons whose signal-to-noise ratio was above that value. A threshold of 1 would mean that signal power = noise power, and any neuron with a higher score is considered to respond above the noise level.

$$\;Signal\;-\;to\;\;\;-\;\;noise\;ratio\;=\frac{\;Signal\;power\;}{\;Noise\;power\;}=\frac{\;Explainable\;variance\;}{\;Total\;\mathrm{var}iance\;\;-\;\;Explainable\;\mathrm{var}iance\;}$$

This technique was used to refine the group of neurons we tested because: **1**/the DH cultures we generate contain both neurons and glia and it is difficult to select neurons based on cell morphology alone. Therefore, the initial data set was likely to contain glial cells, which would be non-responders skewing the final result; **2**/ primary cultures are heterogeneous and therefore neurons can show different responses to the 3 consecutive high K^+^ pulses. Although our exclusion criteria were generous and included neurons showing different patterns of Ca^2+^ transients, we attempted to exclude cells whose 3 pulses were grossly different. The most striking feature of the neurons our Matlab script selected as non-reliable, was the lack of second and third response. Biologically, this effect could be caused by neuronal death or it could be an innate feature of a subgroup of DH neurons. As we were interested in repetitive stimulation of live neurons, we considered both biological reasons for justified. **3/** the Ca^2+^ imaging protocol, as well as the high K^+^ pulse can lead to some neuronal death, which would manifest itself as a lack of a second and/or third responses combined with continuous high Ca^2+^ levels. This type of cells was also considered of low reliability.

From the original group of neurons (WT n = 729 DH neurons and *NCX3*^HOM^ n = 789 DH neurons), 529 WT and 550 *NCX3*^HOM^ remained (derived from WT n = 5 and *NCX3*^HOM^ n = 5 mice, male and female). We repeated the same protocol for DRG neurons. Of the original group of neurons (WT n = 216 and *NCX3*^HOM^ n = 282 DRGs), 179 WT and 197 *NCX3*^HOM^ remained (derived from WT n = 5 and *NCX3*^HOM^ n = 5 mice, male and female). The same Matlab script and threshold (signal-to-noise ratio >1) was used for both DH and DRG neurons.

*Data analysis on refined neurons*. We calculated the following parameters: 1/**baseline** at the start and end of experiment, as well as in between each peak; 2/**peak** values at 90, 180 and 270 s; 3/ **area under the curve (AUC)**, starting at the baseline for each genotype (which ensures that AUC differences are not baseline-dependent) 4/ **tau** – the time constant describing how fast an exponential function decays. Baseline and peak values were detected using Excel and a Matlab script. Area under the curve for each individual cell was calculated using GraphPad prism. The tau value (*τ*) was calculated using a Matlab function for exponential decay and the following equation:

$$f(t)=A\cdot e^{-\;\frac{t}{\tau }}$$

In the above equation, A is the 340/380 ratio at its peak, i.e. at time t = 0. After a period of one time constant (t=), the function reaches e^-1^, which equals 37% of the initial value A. Both WT and *NCX3*^HOM^ responses were normalized prior to analysis, so the final decay value was 0. Tau values above a threshold of 200 s in DH cohort (1 value) and above 100 s in DRG cohort (3 values) were removed.

##### In vivo Ca^2+^ imaging

*Injection of adeno-associated virus 9-GCaMP6s into the lateral parabrachial area*. Under sterile conditions and a stable core body temperature (around 37°C) mice were anesthetized using Isoflurane in oxygen (5% for induction and 2% for maintenance). Analgesia (Carprieve, 0.025 mg; Norbrook Laboratories, Newry, United Kingdom) was administered subcutaneously and the eyes were moistened and protected using eye gel (Viscotears, Liquid Gel, Novartis).

Once mice were positioned in a stereotaxic frame with zygomatic cups and tooth bar/nosecone, a single incision was made to expose the skull. The location of the lateral parabrachial area was identified using lambda and bregma as landmarks. A small hole was drilled in the skull with a dental drill (Ideal Micro-Drill, WPI) and a microinjection setup was used to lower a freshly pulled glass pipette into the lateral parabrachial area. An injection of 800 nL of AAV9.CAG.GcaMP6s.WPRE.SV40 (UPENN Vector Core, AV-1-PV2833,1.1 x 1013 gc/mL) was made slowly (100 nL/minute) into the right lateral parabrachial area. To allow the injected virus bolus to diffuse, the pipette remained in place for 2-5 min post injection after which it was withdrawn slowly. After closing the incision and administering sterile normal saline (0.5 mL at 0.9% - for rehydration), the mice were allowed to recover for at least 11 days.

*In vivo imaging of lamina I projection neurons*. After at least 11 days of recovery post-injection, sufficient GCaMP expression had occurred to visualize lamina I projection neurons using standard single photon microscopy. To anesthetize the mice an initial intraperitoneal dose of urethane, 0.3 mL (12.5% urethane in saline), was given. Mice were then placed onto a heating mat controlled via a rectal probe (Frederick Haer Company, Inc., Bowdoin, ME) and their core body temperature was maintained around 37°C. Additional urethane was administered at 15-min intervals based on depth of anesthesia and reflex activity until surgical depth was achieved.

Once fully anesthetized a laminectomy was performed to expose the underlying dorsal horn of the spinal cord. The back of the mice was shaved, and an incision was made in the skin over the lumbar enlargement. After removing the muscle and connective tissues, the laminae overlying the lumbar enlargement were clipped in a caudal to rostral direction using rongeurs. The dura mater remained intact but was cleaned using normal saline and cotton buds. To visualize the left dorsal horn the exposed spinal segment was stabilized ina partially lateral recumbent position, using spinal clamps attached to the intact vertebrae on either side of the exposure. The exposed spinal segment was covered with silicone elastomer (World Precision Instruments, Ltd, Hitchin, United Kingdom) to ensure a moistened and physiologically intact cord, while also maintaining optical transparency.

The stage with the stabilized mouse was placed under a standard single photon microscope (Eclipse Ni-E FN upright single photon/multiphoton microscope, Nikon, Melville, NY), and the local ambient temperature around the mouse was maintained at 32°C. Time-lapse recordings were taken using a 488-nm argon ion laser line, a 10x dry objective and a 500- to 550-nm bandpass filter. Image acquisition occurred at ~4 Hz and through a fully opened pinhole.

To electrically stimulate lamina I projection neurons, two pin electrodes were placed on either side of the plantar surface of the paw. After a baseline recording of 2 min, a train of 16 square wave pulses of 10 mA and 500 ms were delivered at 0.2 Hz, 0.5 Hz and 1 Hz.

*Image processing and statistical analysis*. Nikon Imaging Software (NIS) Elements AR 0.30.01 was used to align time lapse recordings to a reference frame (Nikon, align application). Further image processing was performed using Fiji/ImageJ version 1.48v. Sta-tistical analysis and graphing were performed using Microsoft Office Excel 2013, IBM SPSS Statistics 23 package, and RStudio 0.99.893. Sample sizes and statistical tests are described in figure legends.

To generate traces of fluorescence over time the cell bodies of lamina I projection neurons were circled in Fiji/ImageJ using the “Freehand selection” tool. Each cell body generated one region of interest (ROI). The fluorescence intensity of pixels located within each ROI was averaged for each time frame, generating the average fluorescence intensity of all cell bodies over time. In addition to the cell body ROIs, a background ROI was generated and averaged in the same way as before. The averaged fluorescence intensity of the background ROI at time *t* was subtracted from each cellular ROI at time *t*. The resulting signal was normalized using the formula

$$\frac{\text{ΔF}}{F}=\frac{F_{t}-F_{0}}{F_{0}}$$

Where F_t_ is the average, background-subtracted fluorescence intensity of a ROI at time *t* and F_0_ is the average, background-subtracted fluorescence intensity of a ROI at a baseline period, prior to the first stimulus. In this manuscript, ΔF/F is expressed as a percentage.

To determine a positive response a threshold for positive responses was set. This was set as follows: 1.2 μ(B_E_) + 2 σ(B_E_). Where B_E_ is the fluorescence intensity over a baseline period prior to each individual event (here defined as a 5 second period, starting 10 s prior to the event of interest) and μ(B_E_) is the average of such signal and σ(B_E_) is the standard deviation of such signal. This resulted in a threshold for a positive response of 20% above the baseline fluorescence intensity + 2 standard deviations.

##### In vitro electrophysiology

*Whole cell patch-clamp recordings of cultured DRG neurons*. Whole cell patch-clamp recordings were performed at room temperature. An Axopatch 200B amplifier was used in conjunction with a Digidata 1500 acquisition system (Molecular Devices). Recording were low-pass filtered at 2 kHz and sampled at 10 kHz. Patch pipettes were pulled from borosilicate glass capillaries (1.5 mm outer diameter, 0.84 mm inner diameter, with filament; World Precision Instruments). Patch pipettes (with 2-5 MΩ tip resistance) were filled with intracellular solution containing (mM): 100 K-gluconate, 28 KCl, 1 MgCl2, 5 MgATP, 10 HEPES, and 0.5 EGTA; pH was adjusted to 7.3 with KOH and osmolarity set to 305 mOsm. The standard extracellular solution used contained (mM): 140 NaCl, 4.7 KCl, 1.2 MgCl2, 2.5 CaCl2, 10 HEPES and 10 glucose; pH was adjusted to 7.3 with NaOH and osmolarity was set to 315 mOsm. Extracellular solution was perfused into the recording chamber (1 ml/min) via a perfusion system. Once whole-cell access was established, resting membrane potential was assessed in bridge mode. Firing properties were assessed in current clamp mode. All current clamp recordings were conducted at a -60 mV holding potential. Input resistance (InR) was calculated from the membrane potential deflection caused by a hyperpolarizing current pulse (80 pA). To define rheobase (minimum current require to elicit and action potential), cells were depolarized by depolarizing current steps (50 ms) of increasing magnitude (D25 pA). Repetitive firing was assessed using depolarizing current injections (500 ms), that increased (D50 pA) from 0 pA-950 pA. The peak membrane potential at 500 ms was analyzed to assess prolonged current induced injection induced depolarization (Data points were excluded only if at 500 ms an action potential occurred). Cells were divided based on diameter, small (<25 μm), medium (25 < 35 μm) and large (>35 μm). Recordings were sampled using pCLAMP11 software (molecular devices) and data analyzed using Clampfit10.7 software (molecular devices).

##### Ex vivo electrophysiology

*Compound action potential recordings*. Compound action potentials (CAPs) were recorded from isolated saphenous nerves from WT or *NCX3*^HOM^ mice. Saphenous nerves were dissected from the inguinal region to the knee. Prior to recording the nerves were desheathed. Each nerve was placed between two suction electrodes and each end pulled through a silicone membrane, to isolate the recording and stimulating sites. The nerves were maintained in a recording chamber (custom built by Dr Roberto De Col), that circulated synthetic interstitial fluid (SIF (mM): 2.0 CaCl_2_, 5.5 Glucose, 10 HEPES, 3.5 KCL, 0.7 MgSO_4_, 123 NaCl, 1.5 NaH_2_PO_4_, 9.5 Na-gluconate, 7.5 Sucrose, pH adjusted to 7.3 using NaOH). Recordings were made at room temperature. Silver wire at either suction electrode allowed for stimulation at one suction electrode and recordings at the other. CAPs were elicited using a constant current stimulator isolator (NL800A, Digitimer), driven by a pulse buffer (NL510A, Digitimer). A supramaximal stimulus (550mA, 150ms) was used to determine CAP amplitude. The nerve of each preparation was measured and used to define conduction velocity. Changes in conduction latency were assessed after 16 consecutive stimuli at 0.25 Hz or 2 Hz, and 240 consecutive stimuli at 2 Hz. All recordings were visualized using an oscilloscope and recorded using a Powerlab 4.0 system in conjunction with LabChart v7.3 software (ADInstruments).

##### In vivo electrophysiology

*Recording form dorsal horn neurons. In vivo* electrophysiology was performed as previously described (Dawes et al., 2018). Both male and female WT (n_female_ = 6, n_male_ = 9), *NCX3*^HET^ (n_female_ = 7, n_male_ = 7) and *NCX3*^HOM^ mice (n_female_ = 7, n_male_ = 6) were used between 8 and 12 weeks old; the experimenter was not blind to genotype. Mice were initially anesthetized with 3.5% v/v isoflurane delivered in 3:2 ratio of nitrous oxide and oxygen. Once areflexic, mice were secured in a stereotaxic frame and subsequently maintained on 1.25% v/v isoflurane for the remainder of the experiment (approximately 3 h in duration). Core body temperature was maintained with the use of a homeothermic blanket and respiratory rate was visually monitored throughout. A laminectomy was performed to expose the L3-L5 segments of the spinal cord; mineral oil was then applied to prevent dehydration. Extracellular recordings were made from deep dorsal horn wide dynamic range (WDR) lamina V/VI neurons with receptive fields on the glabrous skin of the toes using 125 μm 2 MΩ glass-coated tungsten electrodes (Microelectrodes Ltd, Cambridge, UK). Searching involved light tapping of the hind paw whilst manually moving the electrode. All recordings were made at depths delineating the deep dorsal horn laminae, and were classified as WDR on the basis of neuronal sensitivity to dynamic brushing (i.e. gentle stroking with a squirrel-hair brush), and noxious punctate mechanical (15 g) and heat (48°C) stimulation of the receptive field.

The receptive field was then stimulated using a wider range of natural stimuli (brush, von Frey filaments – 1,4,8 and 15 g and heat– 32, 42, 45 and 48°C) applied over a period of 10 s per stimulus and the evoked response quantified. The heat stimulus was applied with a constant water jet onto the center of the receptive field. Ethyl chloride (25 μl) was applied to the receptive field as an evaporative noxious cooling stimulus. Evoked responses to room temperature water (25°C) were subtracted from ethyl chloride evoked responses to control for any concomitant mechanical stimulation during application. Natural stimuli were applied starting with the lowest intensity stimulus with approximately 40 s between stimuli in the following order: brush, von Frey, cold, heat. Receptive fields were determined using a 15 g von Frey. An area was considered part of the receptive field if a response of >30 action potentials over 5 s was obtained. A rest period of 30 s between applications was used to avoid sensitization. Receptive field sizes are expressed as a percentage area of a standardized paw measured using ImageJ (NIH, Bethesda, MD). Electrical stimulation of WDR neurons was delivered transcutaneously via needles inserted into the receptive field after determining responses to natural stimuli. A train of 16 electrical stimuli (2 ms pulses, 0.2 Hz or 0.5 Hz) were applied at three times the threshold current for C-fiber activation. Responses evoked by A- (0–50 ms) and C-fibers (50–250 ms) were separated and quantified on the basis of latency. Neuronal responses occurring after the C-fiber latency band were classed as post-discharge (PD). The input (I) and the wind-up (WU) were calculated as: Input = (action potentials evoked by first pulse) × total number of pulses (16), wind-up = (total action potentials after 16 train stimulus) – Input.

The signal was amplified (x6000), bandpass filtered (low/high frequency cut-off 150/2000 Hz) and digitized at rate of 20 kHz. Data were captured and analyzed by a Cambridge Electronic Design 1401 interface coupled to a computer with Spike2 software v4 (CED, Cambridge, UK) with post-stimulus time histogram and rate functions. One to three neurons were characterized per mouse; in total, 21 neurons were characterized from 15 WT mice, 20 neurons from 14 *NCX3*^HET^ mice, and 20 neurons from 13 *NCX3*^HOM^ mice. *In vivo* electrophysiological procedures were non-recovery; at the end of experiments mice were terminally anesthetized with isoflurane.

*In vivo electromyographic (EMG) recordings to assess the flexion reflex*. Adult female and male WT and *NCX3*^HOM^ (n = 5/group) were used for the flexion reflex recordings. Two standard stainless steel needle recording electrodes (Digitimer, UK) were inserted in the ipsilateral biceps femoris muscle. Sequences of 16 single shocks at 3.2 mA electrical stimulation (DS3 isolated current stimulator, Digitimer, UK) (4ms duration each) were delivered to the mouse paw, using stimulation electrodes that were placed in plantar glabrous skin. Electrical stimulation was delivered at 0.2Hz, 0.5Hz and 1Hz. Data acquisition was performed at Lab Chart 7 software (ADInstruments, New Zealand) and analysis at Spike 2 software (CED, UK) and Prism 9 (GraphPad, USA). The afferent fiber responses to the wind-up protocol were segregated based on conduction velocities (Aβ: 20-100 m/s, Aδ: 2-20 m/s, C: 0.05-2 m/s). The plots depict the number of spikes elicited after each sequential electrical shock. All values represent mean ± SEM. Two-way ANOVA followed by Sidak’s post-hoc test, and non-linear regression analysis were used to compare the responses between wild type and *NCX3* knockout mice, *p < 0.05.

##### Cloning

Cloning of *NCX3*-isoform B into an AAV vector was performed using the In Fusion® Cloning kit (Takara Bio). We used pAAV CAG-GFP viral vector (Addgene, Cat# 37825) as the host plasmid, as well as an insert amplified from *NCX3*-B vector (Origene, Cat# MR211189). In short, we linearized the host plasmid using restriction enzymes BamHI and EcoRI (NEB). The resulting linearized vector was then gel purified. In parallel to this, the *NCX3* insert was amplified from its original plasmid using the following primers:

Forward: GCAAAGAATTGGATCCGCCACCATGGCGTGGTTA,
Reverse: GCTTGATATCGAATTCTTAAACCTTATCGTCGTCATCCTTG.

We included 15bp-long overhangs, which were homologous to the ends of the linearized host vector. We also added a Kozak sequence (GCCACC) at the start of *NCX3* before its original start codon. The resulting *NCX3*-B amplicon, containing the homologous overhangs and the Kozak sequence, was then gel purified. The host plasmid and the insert were then mixed into an In Fusion cloning reaction as described by the manufacturer. The product of the cloning reaction was then used for bacterial transformation using the E coli provided by the manufacturer. After the initial screening of the bacterial colonies, their DNA was extracted using Mini prep kit (Quigen) and sequenced by Source Bioscience Oxford UK. The produced plasmid was then amplified further using MaxiPrep kit (Qiagen) and sent for virus production at the Viral Vector Facility of the Neuroscience centre in Zurich (University of Zurich, UZH). The viruses used in the current study are: ssAAV-1/2-shortCAG-mSlc8a3_myc_FLAG-WPRE-SV40p(A), capsid 1 (custom-made) and ssAAV-1/2-shortCAG-EGFP-WPRE-SV40p(A), capsid 1 (Cat# v587-1).

##### Cell culture

Human embryonic kidney 293 (HEK293) cells were cultured in Dulbeccos’s modified Eagles’s medium with 10% fetal calf serum (TCS Cellworks Ltd) in 24-well plates at 37°C.

For the AAV test experiment, HEK cells were incubated in medium containing 4 μL/ml AAV (stock – 8.5x10^12 vector genomes/mL) for 48 h. On the day of collection, cells were washed with PBS and fixed with 4% PFA for 15 min. This was followed by immunocytochemistry, as described above.

For Ca^2+^ imaging experiments, cells were transfected with either mCherry or NCX3 plasmids, using the Jet PEI reagent (PolyPlus Transfection), following manufacturer’s instructions. Ca^2+^ imaging was performed 24 h post-transfection as described previously. ECF was perfused continuously over the cells. After 60 s of baseline recording, the cells were perfused with ECF, containing1μM Ionomycin for 30 s. This was followed by a 120 s washout.

##### RNA extraction and quantitative real time PCR

For RNA extraction, mice were sacrificed in a CO_2_ chamber. Their DRGs, spinal cord and brain were dissected and snap-frozen in liquid nitrogen, followed by storage at -80°C. On the day of extraction, tissue was mechanically homogenized in Tripure (Roche), treated with chloroform and then subjected to column purification, using a High Pure RNA Tissue kit (Roche). RNA was the eluted in RNAse-free water. cDNA was produced using Transcriptor reverse transcriptase (Roche), random hexamers (Invitrogen) and dNTPs (Roche).

mRNA expression of *NCX3* was achieved using SYBR green. DRG, spinal cord and brain cDNA (5ng) and primers (0.5mM) were added to LightCycler 480 SYBR Green Master mix (Roche) in a 1:1 ratio. 384 well plates (Roche) were then run on a 45 cycle protocol as described by. Primer efficiency and specificity were validated before experimental use. Gene expression was validated against GAPDH as a housekeeping gene, using the delta CT method. Primer sequences are as follows (Michel et al., 2014): *NCX3*-AC_F – GG GCCCCCGCATGGTGGATA, *NCX3*-AC_R – CAGCTTCCTGTCTGTCACTTCTGGA, *NCX3*-B_F - GCATATGGGGAGCTGGAGT, *NCX3*-B_R – GTTCACCAAGGGCAATGAAG.

##### Spinal surgeries

24 female C57BL/6j mice aged 6 - 8 weeks received intraspinal injections using a modified method described by (Foster et al., 2015). Mice were anesthetized with isoflurane, and placed in a stereotaxic frame clamped at the level of T12 and L1 vertebrae. On the right-hand side the intervertebral spaces between T12-T13 and T13-L1 vertebrae were exposed, and a small incision was made through the dura. To minimize swelling and distortion of the spinal cord a small hole was drilled through the lamina of the T13 vertebra, and the dura was opened the same way as in the intervertebral spaces. Intraspinal injection of 300 nL of our *NCX3* virus (described above; 6.3 x 10^7GC/300 nL of diluent; n = 12) or eGFP virus (6.3 x10^7 GC/300 nL of diluent; n = 12) was administered through each of the three holes made through the dura, 400 μm from the midline and 300 μm below the surface of the spinal cord, at a rate of ~30 nl/min with a 10-uL Hamilton syringe attached to a glass micropipette (inner tip diameter ~ 40 μm) using a syringe pump (Pump 11 Elite; Harward Apparatus, Holliston, MA). The virus that each mouse received was randomly assigned and the surgeon was blind to the treatment. To minimize leakage of the injected virus the pipette was left in place for 5 minutes after the completion of each injection. The incision was closed, and mice were allowed to recover. The location of each of the three injections sites described above were chosen to correspond to spinal segments L3, L4 and L5. Animals received perioperative analgesia (buprenorphine 0.3 mg/kg and carprofen 5 mg/kg) and survived for 2 weeks.

### Quantification And Statistical Analysis

#### Human experiments

##### Quality control filtering of data for individuals

We excluded individuals representing phenotypic outliers, defined as a WUR > 6 (primarily caused by very small numerical pain ratings for the single stimulus, e.g., 0.001). We excluded individuals with more than 5% missing SNP genotypes and those that failed the X- or Y-chromosome sex concordance check based on the chip data (sex estimated from X-chromosome heterozygosity or Y chromosome call rate not matching recorded sex information). 4 individuals in the discovery cohort failed the sex concordance check and were excluded. The chip genotype data was used to estimate the Native American, European and African ancestry of individuals, using the program ADMIXTURE (key resources table). African ancestry outliers (i.e. individuals with >30% African ancestry) were removed. Individuals with high relatedness (i.e. those with IBD > 0.1 estimated from the chip data) were also removed. Genetic principal components (PCs) were calculated on this set of unrelated individuals, and outliers for any of the top PCs were excluded. PCs were recalculated each time after the removal of outliers, and this procedure repeated until no further outliers were observed. After applying all quality control filters, 996 individuals were retained for further analyses.

##### SNP genotype imputation

The chip genotype data were phased using SHAPEIT2 (key resources table). IMPUTE2 (key resources table) was then used to impute genotypes at untyped SNPs using genome sequence information from the 1000 Genomes Project (phase 3), comprising phased data for 2,504 individuals across the world, including a total of 77,818,332 autosomal and X-chromosome variant positions. We excluded positions that are monomorphic in the 1000 Genomes Latin American samples (CLM, MXL, PEL and PUR), leading to 11,218,392 SNPs being imputed in our data set. Of these, we removed 64,681 SNPs based on the following criteria that may indicate poor imputation and/or genotyping quality: either (i) imputation quality scores <0.4, or (ii) low concordance value (<0.7) with chip genotypes, or (iii) alarge gap between info (a cross-validation metric implemented in IMPUTE2 (key resources table), indicating imputation certainty) and concordance values (info_type0-concord_type0>0.1). In the latter two cases, these SNPs were also excluded from the chip data set. The IMPUTE2 genotype probabilities at each locus were converted into most-probable genotypes using PLINK (key resources table) (at the default setting of >0.9 certainty). Finally, SNPs with the proportion of samples with uncalled genotypes >5% and minor allele frequency <1% were also excluded. The final imputed data set contained genotypes for 9,616,058 SNPs.

For each imputed index SNP, a nearby SNP is chosen among the SNPs on the genotyping chip which is in the same LD block as the index SNP and has the smallest P-value among all such chip SNPs. Various quality measures are presented for both the index SNP and this chip genotyped SNP, and the chip genotype calling plots produced by Illumina GenomeStudio v2.0 are also provided (Table S2). All the measures were within their acceptable range.

We verified the accuracy of imputation in the final data set, especially for Native American variants, as this continental ethnic group is a major source population for Latin Americans but are not represented independently in the 1000 Genomes cohort. First, we estimated the imputation accuracy of the imputed data set using the median info score. The overall median info score was 99.2%. It is also high (99.5%) for “European” variants (defined as variants with MAF > 20% in highly European individuals and MAF < 5% in highly Native American individuals) as well as for “Native American” variants (99.3%) and “African” variants (90.9%). For the sake of comparison, the rare variants (MAF < 5% in Colombians; ~30% of the imputed data set) have a median info score of 97.4%. We have therefore concluded that imputation was reliable for variants of all major ethnicities, and was high overall for the entire imputed dataset including rare variants as observed from the median values (As SNPs with MAF < 1% in the AMR reference population or in the Colombian samples are excluded, we consider a threshold of MAF < 5% for ‘rare’ SNPs.)

In addition, we sought to verify the quality of imputed genotypes by comparing with two independently sequenced datasets. A set of Native American samples collected for a previous study (Chacón-Duque et al., 2018) were sequenced at high coverage and variants filtered. We calculated the concordance for these samples as the proportion of imputed genotypes that match the sequence data exactly. The overall median concordance was high (99.4%) and remained high in sub-categories: 98.9% for SNPs common in Native American samples (MAF > 5% in that population) and 99.8% for rare SNPs in Native American samples (MAF < 5%). For another set of sequenced European samples, the average concordance for imputed SNPs were equally high at 99.2%.

##### Association analyses

We used PLINK v1.9 (key resources table) to perform a GWAS using multiple linear regression of WUR on the imputed genotype data, with sex, age, QIDS score, and the 4 leading genetic PCs as covariates. The number of PCs to be included in the regression model was determined by inspecting the proportion of variance explained and by checking scree and PC scatter plots (Figures S1D and S1E). The Q-Q plot showed no evidence of inflation (Figure S1F), the genomic control factor lambda being ~1.03, showing no evidence of population structure.

Association tests for the index SNPs detected in this cohort for WUR were also tested against other QST phenotypes to check for any shared association signals (Table S3). No associations were genome-wide significant.

##### Statistical power

Power calculations for GWAS conducted in this cohort was published previously in the protocol paper (Schmid et al., 2019), which provided both estimated power for various parameter values and an R code to conduct such calculations (key resources table). For the current sample size of the study (~1000) and the proportions of trait variance (R^2^) explained by the index SNPs as observed here (Table S1), we estimated 54% and 96% power to detect genome-wide significant associations, for R^2^ = 3% and 5% respectively.

##### Robust association analyses

Considering the skewed trait distribution of WUR (Figure S1C), robust association analyses were carried out in two different ways to validate the associations of the index SNPs seen in the primary cohort. First, a robust linear regression model (function lmrob implemented in the R package robustbase) was used for the WUR phenotype, which does not make the traditional assumptions such as normal distribution or homoscedasticity, instead of the classical linear regression model implemented in PLINK.

Second, the WUR trait values were transformed using the Box-Cox power transformation (function bcPower implemented in the R package car), to make the distribution closer to a normal distribution. The parameter of the transformation was tuned using the Lil-liefors test (function lillie.test implemented in the R package nortest) to pick the value that provided the most consistency with the normal distribution. The value of -1.1 was chosen in this process. The transformed trait values were then used in the classical linear regression model.

P values from the two analyses for the three index SNPs are presented in Table S4. In general, these robust association analyses are more conservative and therefore yield slightly weaker p values.

##### Replication analysis: Second Colombian cohort

To evaluate replication of the GWAS signal observed in the primary Colombian cohort, we examined a second, smaller Colombian cohort which was collected (between Jan 2017 and July 2019) at the same location by the same rater. This cohort was phenotyped for wind-up in the same way as the primary cohort. Genotyping, quality control of the genetic data, and imputation was also performed in the same way. A total of 317 individuals were used in this replication GWAS analysis. No individuals failed the sex concordance check.

The average ancestry of these individuals was estimated as: 30% Native American, 58% European and 12% African, very similar to the primary cohort. The genomic control factor lambda was 1.01, showing no evidence of population structure. P values of the three index SNPs obtained in the primary cohort were extracted and presented in Table S1.

##### Replication analysis: OPPERA cohort

To evaluate replication of the GWAS signal observed in the Colombian data, we examined association for a wind-up phenotype examined in the diverse OPPERA (Orofacial Pain Prospective Evaluation and Risk Assessment) study (Greenspan et al., 2011). This is a population-based study that recruited individuals aged 18 to 44, with a diverse ethnic background, at four sites (Buffalo, New York; Gainesville, Florida; Baltimore, Maryland and Chapel Hill, North Carolina). According to self-reported ethnicity, the cohort included White, African-American, Hispanic, Asian, ‘Native Hawaiian or other Pacific Islander’, and ‘American Indian or Alaskan Native’ participants among others (Slade et al., 2011).

OPPERA focuses on temporomandibular disorders (TMD) and has collected over 200 pain phenotypes and pain related comorbidities. Here we selected non-TMD controls that had been assessed for temporal summation (the difference in pain rating between the average of a train of 10 stimuli and an initial single stimulus) on a Visual Analogue Scale (VAS 0–100), using a 256 mN probe (Greenspan et al., 2011). The OPPERA publication established the protocol for measuring wind-up as a difference between the pain ratings, whereas the previously published protocol for the Colombian cohort defined wind-up as a ratio (Schmid et al., 2019), in all cases agnostic of any genetic association studies. Therefore, we decided to continue to use the phenotypes as defined in their corresponding protocols, despite slight differences in definition between the two cohorts.

A total of 2868 control individuals had been genotyped with the Illumina HumanOmni 2.5 Exome Bead Chip platform (Smith et al., 2019). The cohort consisted of a diverse set of participants with White, Black and other ethnicities. Quality control of the genotypes was performed, and these were subsequently phased using SHAPEIT2 (key resources table) and imputed with the 1000 Genomes Phase 3 panel using IMPUTE2 (key resources table).

For the GWAS analysis of the trait, the study used a linear regression model with age, sex, recruitment center, 3 PCs, and body mass index as covariates. P values of imputed SNPs in the association regions observed in the primary GWAS (Table S1) were extracted. The genomic inflation factor (lambda) was = 1.02, showing no evidence of population structure. As the primary and replication cohorts differ substantially in their ethnic composition of participants, we aimed for a gene-level replication of association, instead of replication of individual index SNPs. The GCTA-fastBAT test was implemented for each of the genes reported in Table S1, combining the SNP-level p values into a gene-level p value (key resources table). The rank of those p values out of the 24418 genes tested are also provided.

#### Mouse experiments

##### Data analysis and p values

Data are shown as means ± SEM. Statistical tests were carried out in GraphPad prism 9.0. All data were tested for normality using visual inspection and the Kolmogorov-Smirnov test. When only two experimental groups were compared, independent t-tests were performed. When more than two experimental groups were tested, we performed one-way ANOVA with the respective multiple comparisons tests. For formalin experiments and *in vivo* electrophysiology, when analyzing data over time, we used two-way ANOVA with Dunnett’s or Bonferroni multiple comparisons test.

## Supplementary Material

Supplemental information can be found online at https://doi.org/10.1016/j.neuron.2022.05.017.

Supplementary Material

## Data Availability

Summary statistics from the primary GWAS analysis have been deposited at GWAS central, to be available at http://www.gwascentral.org/study/HGVST5028 during their summer 2022 data release. Data relating to investigations in mice are available on request to D.L.B. All other data are available in the main text or the supplemental information. All original code has been deposited at Github and is publicly available as of the date of publication. DOIs are listed in the key resources table. Any additional information required to reanalyze the data reported in this work paper is available from the lead contact upon request.

## Highlights

- Significant genetic association between human pain wind-up and the NCX3 locus
- NCX3 null mice show hypersensitivity in inflammatory and neuropathic pain models
- NCX3 null dorsal horn spinal neurons show increased windup and intracellular Ca^2+^
- Virally mediated spinal overexpression of *NCX3* reduces pain-related behavior in mice

## In brief

Repetitive noxious stimuli increase pain perception (wind-up). Trendafilova et al. demonstrate significant genetic association between the sodium-calcium exchanger NCX3 and human wind-up. Mice lacking NCX3 show increased pain-related behavior and spinal hyper-excitability due to slowed Ca^2+^ clearance. Enhanced spinal expression of *NCX3* reduced pain in mice, suggesting therapeutic potential.
