# Supplementary material for: Sodium-calcium exchanger-3 regulates pain “wind-up”: From human psychophysics to spinal mechanisms
Source: Neuron. Author manuscript; Available in PMC 2022 Aug 29. (PMC7613464; doi:10.1016/j.neuron.2022.05.017)
Supplement: Supplementary file 1 [file EMS152407-supplement-Supplementary_Material.pdf]

Neuron, Volume *110*

Supplemental information

Sodium-calcium exchanger-3 regulates pain “wind-up”: From human psychophysics to spinal mechanisms

Teodora Trendafilova, Kaustubh Adhikari, Annina B. Schmid, Ryan Patel, Erika Polgár, Kim I. Chisholm, Steven J. Middleton, Kieran Boyle, Allen C. Dickie, Evangelia Semizoglou, Jimena Perez-Sanchez, Andrew M. Bell, Luis Miguel Ramirez-Aristeguieta, Samar Khoury, Aleksandar Ivanov, Hendrik Wildner, Eleanor Ferris, Juan-Camilo Chacón-Duque, Sophie Sokolow, Mohamed A. Saad Boghdady, André Herchuelz, Pierre Faux, Giovanni Poletti, Carla Gallo, Francisco Rothhammer, Gabriel Bedoya, Hanns Ulrich Zeilhofer, Luda Diatchenko, Stephen B. McMahon, Andrew J. Todd, Anthony H. Dickenson, Andres Ruiz-Linares, and David L. Bennett

Supplementary items

Index SNP	Chromosomal Region	Alleles (minor/ major)	P-value (from discovery cohort)	Candidate gene	Beta	R^2	Replication *P*-value from second Colombian cohort	OPPERA gene-level Replication *P*-value (rank)	Minor Allele Frequency			
									Europeans (IBS)	Native Americans	Africans (YRI)	Colombians
rs58886412	1q21.3	C/G	1×10^{-8}	Intergenic (*S100A16*)	0.43	3%	6×10^{-3}	0.31 (8101)	2%	2%	18%	4%
rs115943328	14q24.2	C/T	1×10^{-11}	*SLC8A3 (NCX3)*	0.45	5%	3×10^{-5}	3×10^{-8} (1)	0%	0%	18%	2%
rs73240416	14q21.2	G/A	8×10^{-9}	*LINC00871*	0.67	3%	0.013	0.005 (94)	0%	0%	31%	2%

Supplementary Table 1. Features of SNPs most strongly associated with WUR on Chromosomes 1 and 14 in the Colombian cohort. (Related to Figure 1) The two alleles are consecutively minor and major alleles. R^2 refers to the proportion of trait variance explained by the SNP beyond that explained by the covariates. The primary association P-value, Beta and R^2 are those obtained in the initial (discovery) Colombian cohort. Due to its internal heterogeneity (and differentiation from the Colombian sample), replication P-values for the OPPERA cohort refer to a gene-level association test. The rank of those P-values out of the 24,418 genes tested are also provided. Minor allele frequency for the Europeans (Iberians, IBS) and Africans (Yoruba, YRI) are obtained from the 1000 Genomes Project. Native American data are from (Chacón-Duque *et al.*, 2018). Colombians refers to the individuals examined here in the discovery cohort.

Chrom. region	Type	SNP	Distance to index SNP (KB)	MAF	HWE P-value	R^2 with index SNP	Imputation quality metric*	Genotype calling plot for chip SNP
1q21.3	Index (imputed) SNP	rs58886412	-	4%	0.83	-	0.940	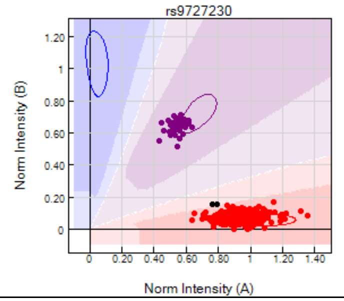
	Chip genotyped SNP	rs9727230	4.5	4%	0.84	0.97	0.980	
14q24.2	Index (imputed) SNP	rs115943328	-	2%	0.09	-	0.988	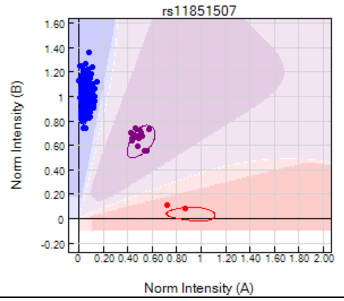
	Chip genotyped SNP	rs11851507	28.5	2%	0.06	0.42	0.998	
14q21.2	Index (imputed) SNP	rs73240416	-	2%	0.14	-	0.994	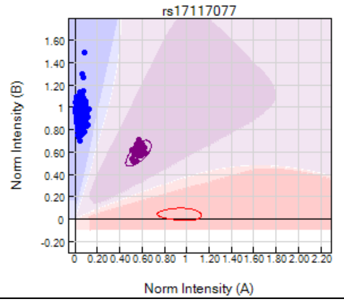
	Chip genotyped SNP	rs17117077	8.5	2%	0.15	0.97	0.998	

Supplementary Table 2. Various quality measures for the three index SNPs and their nearby chip genotyped SNPs. (Related to Figure 1) For each imputed index SNP, a nearby SNP is chosen among the SNPs on the genotyping chip which is in the same LD block as the index SNP and has the smallest P-value among all such chip SNPs. Various quality measures are presented for both the index SNP and this chip genotyped SNP.

MAF: Minor allele frequency.

HWE: Hardy-Weinberg equilibrium.

For the chip genotyped SNP, the distance to their index SNP (in KB), and their R^2 with the index SNP is also provided. Their genotype calling plot, as produced by Illumina GenomeStudio v2.0 software, is also provided.

* For each imputed SNP, the info metric reported by IMPUTE2 is provided. For each chip genotyped SNP, the concordance metric reported by IMPUTE2 is provided, which performs leave-one-out imputation and checks concordance with the true genotype

Phenotype	rs58886412 (1q21.3)	rs115943328 (14q24.2)	rs73240416 (14q21.2)
Cold detection threshold (CDT)	0.487	0.066	0.758
Warm detection threshold (WDT)	0.469	0.083	0.316
Thermal sensory limen (TSL)	0.623	0.195	0.229
Heat pain threshold (HPT)	0.785	0.473	0.842
Mechanical pain threshold (MPT)	0.367	0.699	0.791
vibration detection threshold (VDT)	0.728	0.573	0.034
Flare area	0.224	0.056	0.121
Brush-evoked allodynia	0.153	0.016	0.760
Punctuate hyperalgesia	0.010	0.108	0.548

Supplementary Table 3. Association results for the three index SNPs with other QST phenotypes in the primary Colombian cohort. (Related to Figure 1) The *P*-values for each of the three index SNPs for each of the QST phenotypes are presented.

Chromosomal region	SNP	Robust linear regression P-value	Box-Cox transformed trait P-value
1q21.3	rs58886412	5×10^{-7}	7×10^{-7}
14q24.2	rs115943328	5×10^{-10}	1×10^{-8}
14q21.2	rs73240416	4×10^{-6}	3×10^{-5}

Supplementary Table 4. Robust test and transformed phenotype association results for the three index SNPs in the primary Colombian cohort. (Related to Figure 1) The P-values for each of the three index SNPs are presented for two methods. First, a robust linear regression model was used for the WUR phenotype. Second, the WUR phenotype was transformed using the Box-Cox power transformation to make the distribution closer to a normal distribution and transformed trait values were then used in the classical linear regression model.

	Small (<25µm)			Medium (25<35µm)			Large (>35µm)		
	$NCX3^{WT}$	$NCX3^{HOM}$	Test	$NCX3^{WT}$	$NCX3^{HOM}$	Test	$NCX3^{WT}$	$NCX3^{HOM}$	Test
No. Cells	35	29		33	32		14	13	
Cm (pF)	14.21 (±0.73)	11.62 (±0.66)	MW, *P*=0.01, *	28.55 (±1.17)	23.94 (±0.94)	MW *P*=0.0017 **	44.57 (±2.00)	40.85 (±2.13)	uTt *P*=0.213 n.s.
InR (MΩ)	301.30 (±21.4)	618.20 (±67.4)	MW, *P*<0.0001, ****	96.02 (±10.5)	197.10 (±24.5)	MW, *P*<0.0001, ****	31.25 (±4.92)	69.90 (±15.01)	MW, *P*=0.002, **
RMP (mv)	-53.78 (±1.43)	-45.88 (±1.52)	MW, *P*=0.0002, ***	-63.03 (±0.80)	-61.70 (±0.95)	uTt, *P*=0.287, n.s	--65.61 (±0.88)	-65.12 (±0.97)	uTt, *P*=0.71, n.s

Supplementary Table 5. Passive electrophysiological properties of DRG neurons isolated from $NCX3^{WT}$ and $NCX3^{HOM}$ mice. (Related to Supplementary Figure 6 and Figure 3,4 and 6) Mean (±SEM), Cm = Membrane Capacitance, InR = Input Resistance, RMP = Resting Membrane Potential, MW = Mann Whitney Test, uTt = unpaired T-test.* - *P*<0.05, ** - *P*<0.001, *** - *P*<0.0001, **** - *P*<0.0001

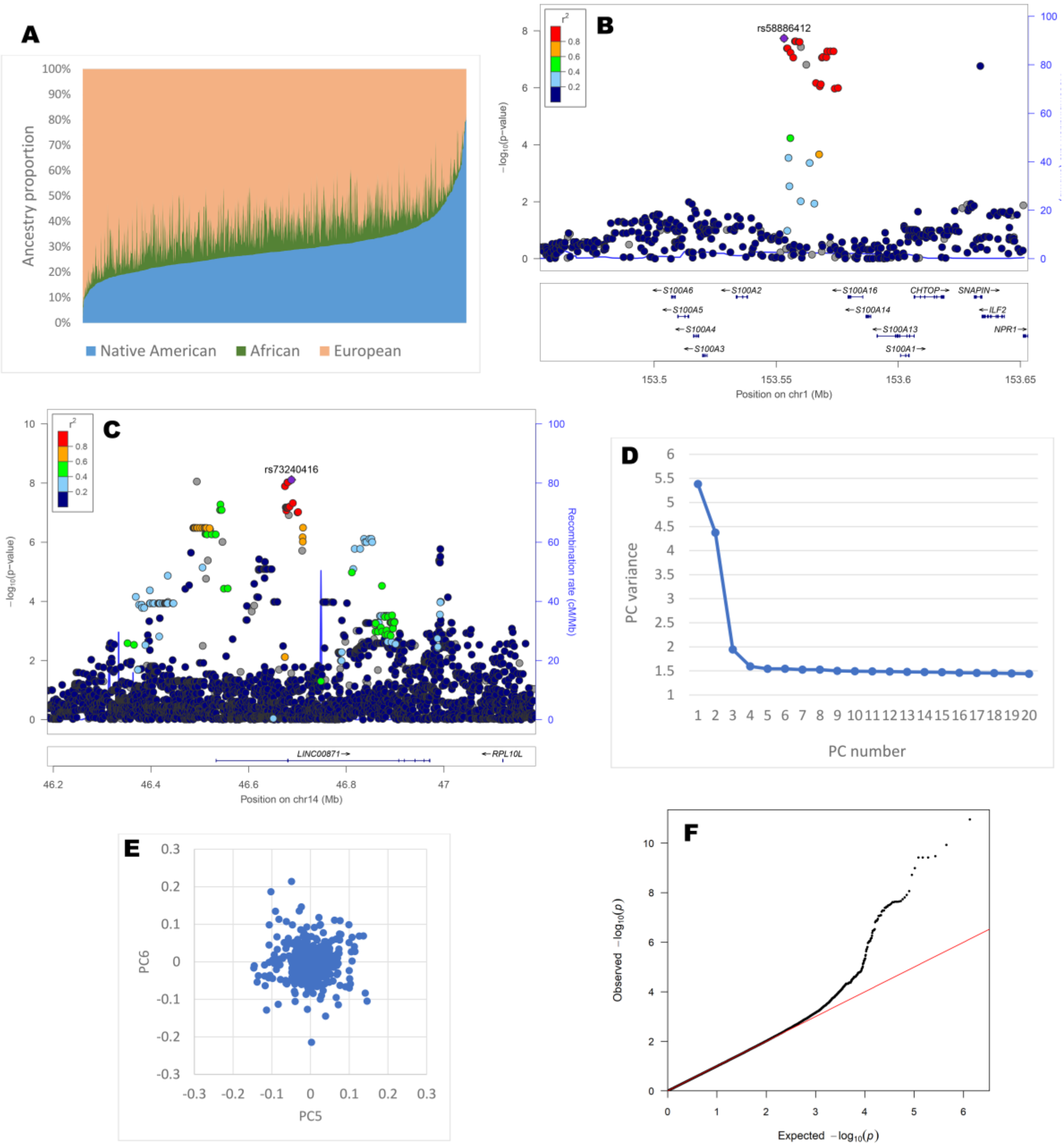
A
Ancestry proportion
100%
90%
80%
70%
60%
50%
40%
30%
20%
10%
0%
Native American
African
European
B
rs58886412
r2
0.8
0.6
0.4
0.2
−log10(p−value)
S100A6
S100A2
S100A5
S100A4
S100A3
S100A16
S100A14
S100A13
S100A1
CHTOP
SNAPIN
ILF2
NPR1
153.5
153.55
153.6
153.65
Position on chr1 (Mb)
C
rs73240416
Recombination rate (cM/Mb)
LINC00871
RPL10L
46.2
46.4
46.6
46.8
47
Position on chr14 (Mb)
D
PC variance
PC number
E
PC6
PC5
F
Observed −log10(p)
Expected −log10(p)

Supplementary Figure 1. Supporting results from human genetic association studies in the Colombian cohort. (Related to Figure 1) **A.** Continental ancestry estimates of the Colombian participants, using three ancestral components as reference: Native American, European, and African. **B.** LocusZoom plot of the association results around the index SNP rs58886412 in chromosome 1q21.3. Negative logarithm of the GWAS P-values are plotted on the Y axis. **C.** LocusZoom plot of the association results around the index SNP rs73240416 in chromosome 14q21.2. **D.** Scree plot showing the variance of the top 20 Genetic principal components (PCs) obtained using chip genotyped SNPs. Based on this plot and the pairwise scatterplots of PCs, the top 4 PCs were chosen to be indicative of population substructure and to be used as covariates in the GWAS. **E.** Scatterplot of PCs 5 & 6, indicating that no residual substructure is visible. **F.** Q-Q plot of GWAS P-values for WUR in the Colombian cohort. Negative logarithm of the observed and expected P-values are plotted on the Y and X axis respectively. The diagonal red line is provided for reference. The genomic inflation factor λ was 0.9986.

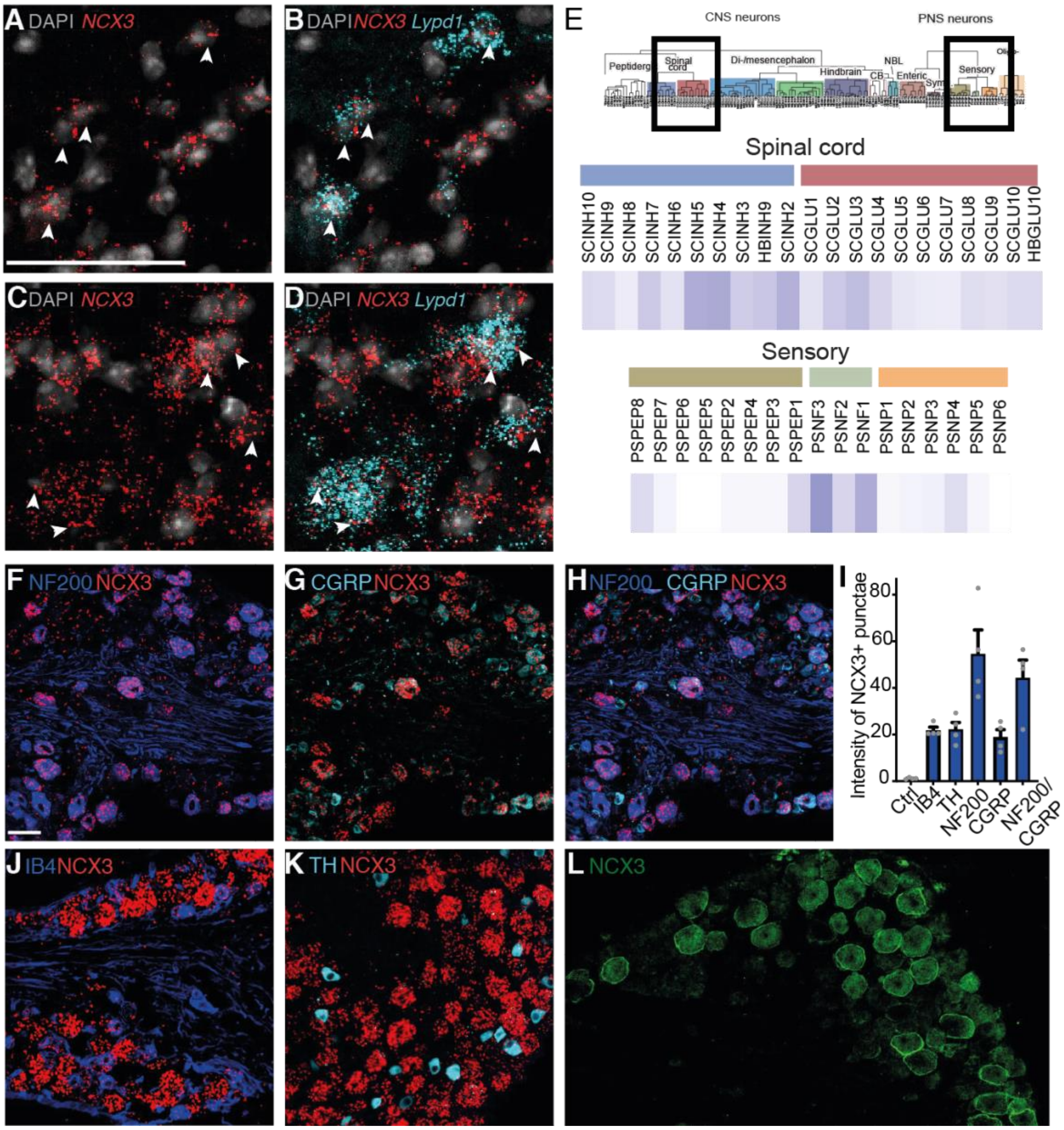
A DAPI NCX3
B DAPI NCX3 Lypd1
C DAPI NCX3
D DAPI NCX3 Lypd1
E
CNS neurons
PNS neurons
Spinal cord
SCINH10 SCINH9 SCINH8 SCINH7 SCINH6 SCINH5 SCINH4 SCINH3 HBINH9 SCINH2 SCGLU1 SCGLU2 SCGLU3 SCGLU4 SCGLU5 SCGLU6 SCGLU7 SCGLU8 SCGLU9 SCGLU10 HBGLU10
Sensory
PSPEP8 PSPEP7 PSPEP6 PSPEP5 PSPEP2 PSPEP4 PSPEP3 PSPEP1 PSNF3 PSNF2 PSNF1 PSNP1 PSNP2 PSNP3 PSNP4 PSNP5 PSNP6
F NF200 NCX3
G CGRP NCX3
H NF200 CGRP NCX3
I
Intensity of NCX3+ punctae
0 20 40 60 80
Ctrl IB4 TH NF200 CGRP NF200/CGRP
J IB4 NCX3
K TH NCX3
L NCX3

Supplementary Figure 2. **Expression of *NCX3* in DH projection neurons and dorsal root ganglia.** (Related to Figure 2) **A-D.** Qualitative example image showing multiplex ISH for *NCX3* and *Lypd1* (projection neuron marker) mRNA in the spinal dorsal horn. **E.** Data from *(Zeisel* et al.*, 2018)* derived from http://mousebrain.org/adolescent showing expression levels of *NCX3* in spinal cord and sensory neurons please see **F-K.** Representative images of NCX3 in-situ hybridisation (ISH) combined with immunofluorescence for DRG neuronal markers (IB4, CGRP, NF200 and TH). **I.** Quantification of the 5 images from F-K as average intensity (AU). **L.** Qualitative example image showing NCX3 protein expression in the DRG. (3 images per animal, n=5 animals). Scale bar = 50 µm.

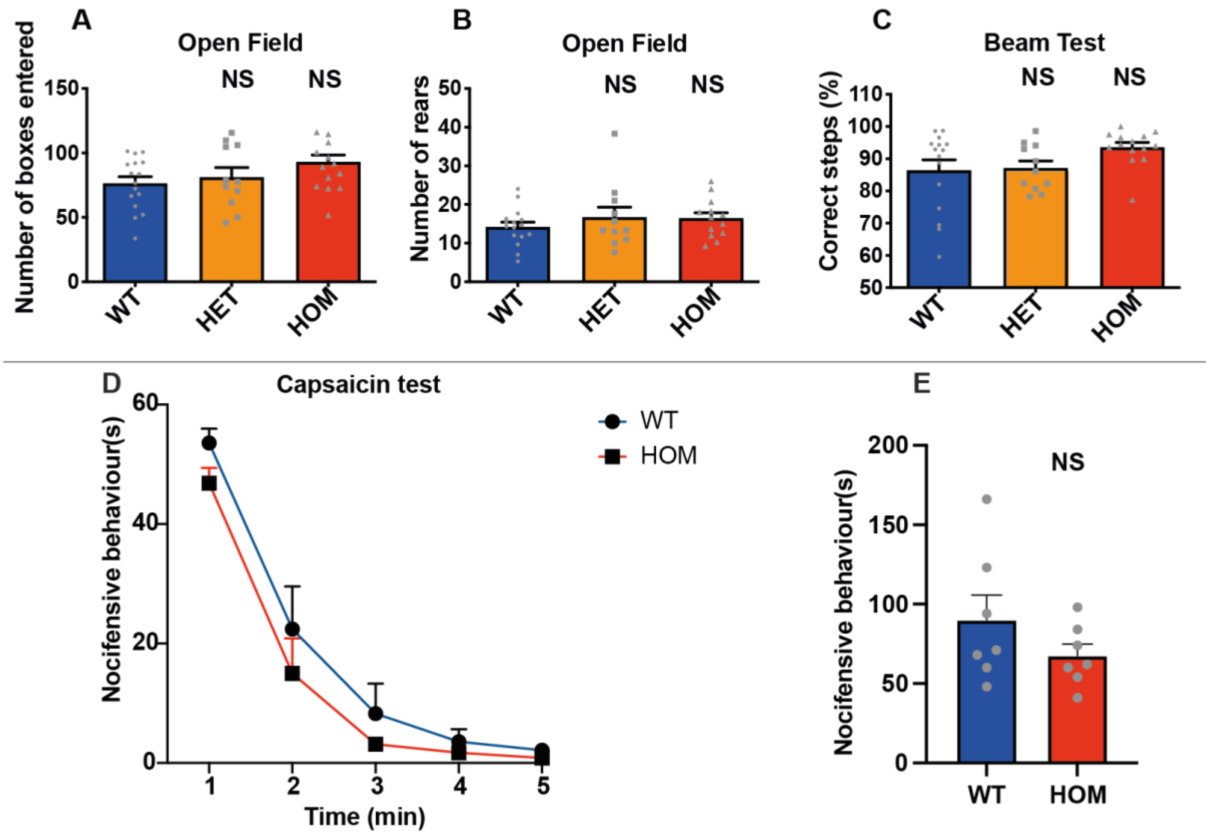
A
Open Field
NS
NS
Number of boxes entered
150
100
50
0
WT
HET
HOM
B
Open Field
NS
NS
Number of rears
50
40
30
20
10
0
WT
HET
HOM
C
Beam Test
NS
NS
Correct steps (%)
110
100
90
80
70
60
50
WT
HET
HOM
D
Capsaicin test
Nocifensive behaviour(s)
60
40
20
0
1
2
3
4
5
Time (min)
WT
HOM
E
NS
Nocifensive behaviour(s)
200
150
100
50
0
WT
HOM

Supplementary Figure 3. Lack of motor phenotypes in NCX3^{HET} and NCX3^{HOM} mice. (Related to Figure 3) **A-C** Motor/anxiety/balance behaviour tests. Data are mean ± SEM, WT n=16, NCX3^{HET} n=11, NCX3^{HOM} n=13. **D and E.** Response over time during the first 5 minutes post capsaicin injection. **B.** Mean results from the first 5 minutes post-injection. Data are mean ± SEM, WT n=7, NCX3^{HOM} n=7. Data analysis: Significance shows comparison to WT (NS P>0.05). Data analysis – One-Way ANOVA, Dunnett's multiple comparisons test (A-C). Two-Way ANOVA, Sidak multiple comparisons test (D); Unpaired T-test (E).

-

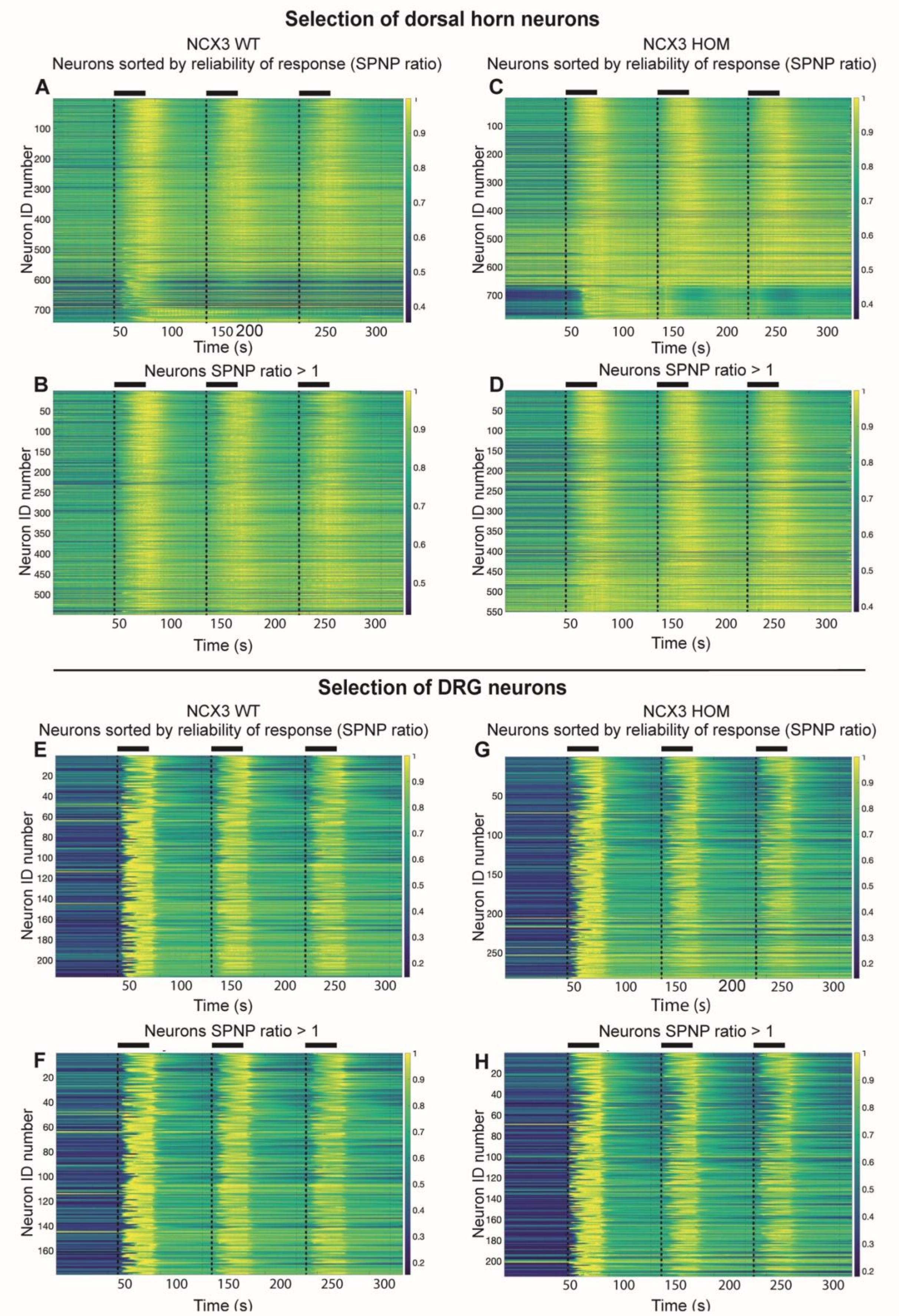
Selection of dorsal horn neurons
NCX3 WT
Neurons sorted by reliability of response (SPNP ratio)
NCX3 HOM
Neurons sorted by reliability of response (SPNP ratio)
A
C
Neuron ID number
Time (s)
B
Neurons SPNP ratio > 1
D
Neurons SPNP ratio > 1
Selection of DRG neurons
NCX3 WT
Neurons sorted by reliability of response (SPNP ratio)
NCX3 HOM
Neurons sorted by reliability of response (SPNP ratio)
E
G
F
Neurons SPNP ratio > 1
H
Neurons SPNP ratio > 1

Supplementary Figure 4. Sorting of cultured neurons based on calcium imaging response reliability. (Related to Figure 4) **A, C.** Raster plots showing the Ca^{2+} responses of DH neurons to 50mM K^+ pulses (each pulse starting at 60, 150 or 240 seconds and lasting for 30 seconds). Beginning and duration of each K^+ pulse - described with dashed vertical lines and black bars. Neurons were sorted based on similarity between their three Ca^{2+} responses (reliability, as measured by Signal power/Noise power ratio). **B and D.** Raster plots showing the dorsal horn neurons from A (WT) and B ($NCX3^{HOM}$), respectively, which displayed reliability of response (SPNP ratio) higher than 1. **E, G** Raster plots showing the Ca^{2+} responses of DRG neurons to 50mM K^+ pulses. **F, H.** Sorted DRG neurons, SPNP ratio>1. Plot colour scheme – blue to yellow corresponds to low to high Ca^{2+} level.

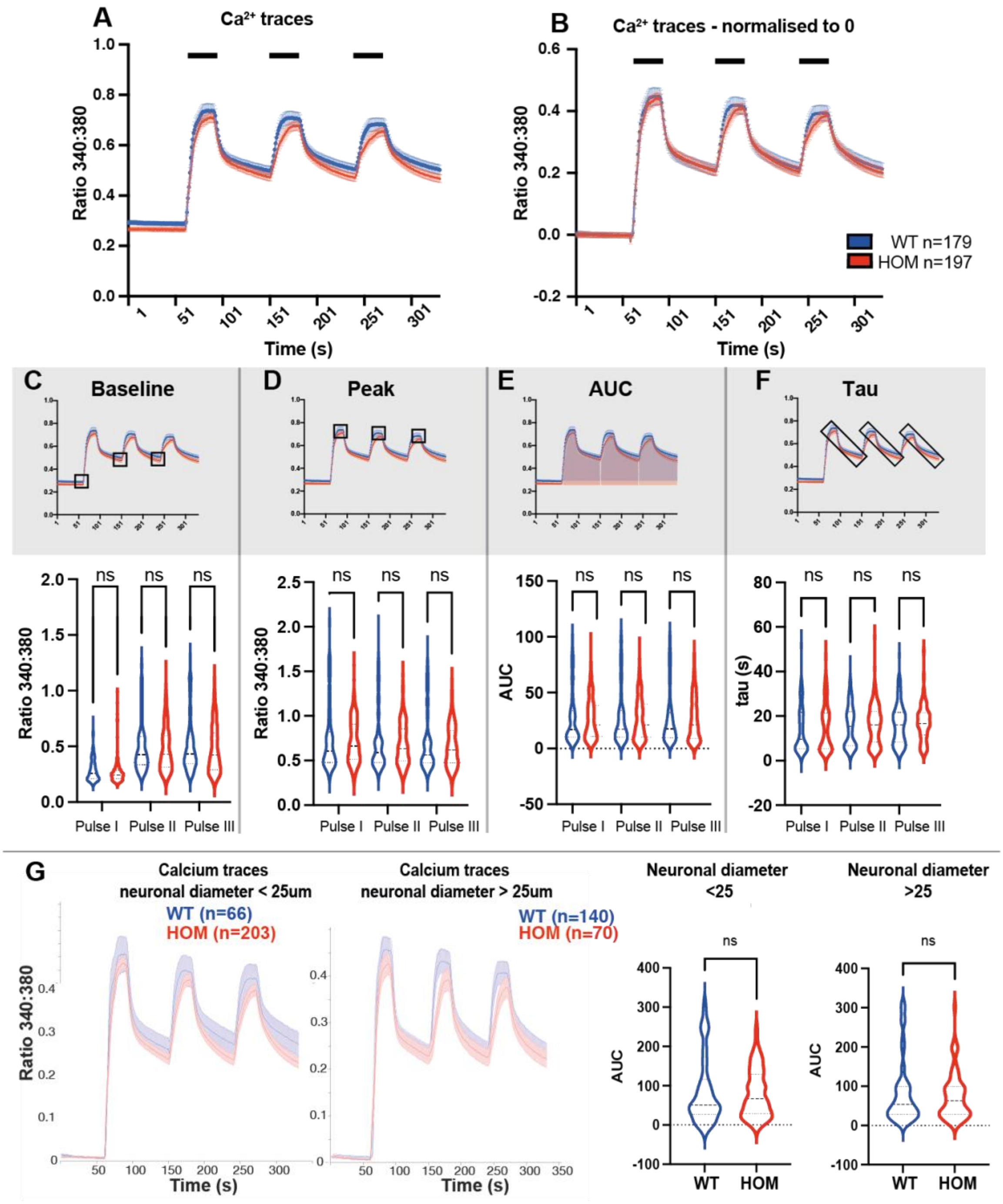
A
Ca²⁺ traces
Ratio 340:380
Time (s)
B
Ca²⁺ traces - normalised to 0
Ratio 340:380
Time (s)
WT n=179
HOM n=197
C
Baseline
D
Peak
E
AUC
F
Tau
ns
Pulse I
Pulse II
Pulse III
Ratio 340:380
AUC
tau (s)
G
Calcium traces
neuronal diameter < 25um
WT (n=66)
HOM (n=203)
Calcium traces
neuronal diameter > 25um
WT (n=140)
HOM (n=70)
Ratio 340:380
Time (s)
Neuronal diameter
<25
Neuronal diameter
>25
AUC
WT
HOM

Supplementary Figure 5. No change to Ca^{2+} dynamics in cultured *NCX3*^{HOM} DRG neurons. (Related to Figure 4) **A.** Ca^{2+} traces during 3 K^+ pulses (mean ± SEM). **B.** Traces after baseline normalisation. **C-F**. Assessed properties of the calcium imaging traces, top panels in grey are schematics explaining the properties compared in panels below shown as violin plots; **C.** Baselines at 60 seconds (Pulse I), 150 seconds (Pulse II) and 240 seconds (Pulse III). **D.** Peaks at 90 seconds (Pulse I), 180 seconds (Pulse II) and 270 seconds (Pulse III). **E.** Area under the curve (AUC) for 60 to 150 seconds (Pulse I), 150-240 seconds (Pulse II) and 240-330 seconds (Pulse III). **F.** Exponential decay time constant (tau) for 90 -150 seconds (Pulse I), 180 to 240 seconds (Pulse II) and 270 to 330 seconds (Pulse III). G. Sub-group analysis based on neuronal cell diameter shown as violin plots. Significance was assessed with One-Way ANOVA, Tukey test, NS P>0.5. n=179 WT and 197 *NCX3*^{HOM} DRG neurons, from WT n=5 and *NCX3*^{HOM} n=5 mice (A-F). Student's T-test (G).

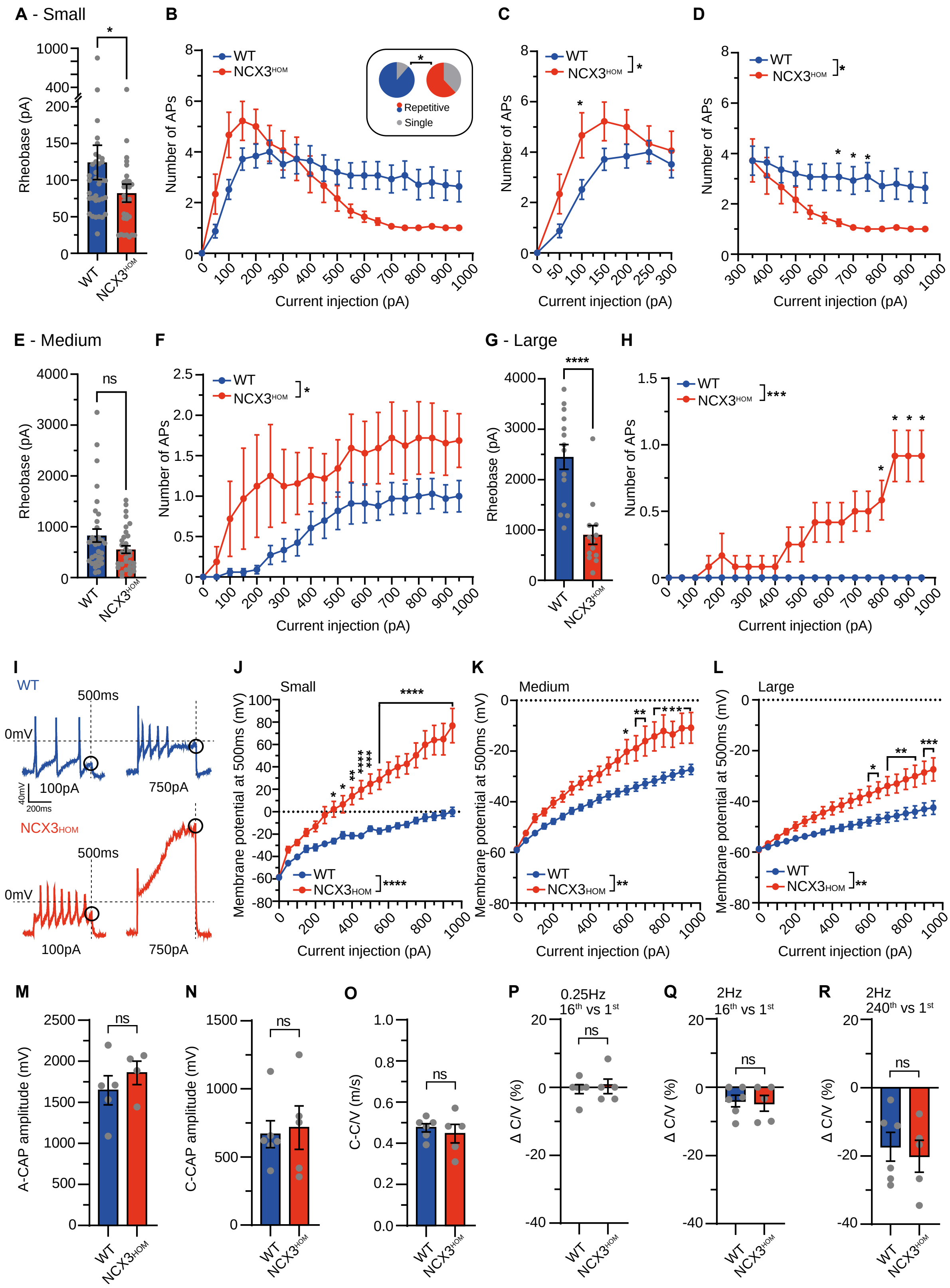

A - Small
B
C
D
E - Medium
F
G - Large
H
I
J
K
L
M
N
O
P
Q
R
Rheobase (pA)
Number of APs
Current injection (pA)
WT
NCX3HOM
Repetitive
Single
Small
Medium
Large
500ms
0mV
100pA
750pA
40mV
200ms
Membrane potential at 500ms (mV)
A-CAP amplitude (mV)
C-CAP amplitude (mV)
C-C/V (m/s)
Δ C/V (%)
0.25Hz
16th vs 1st
2Hz
16th vs 1st
2Hz
240th vs 1st
ns

Supplementary Figure 6: Soma excitability, but not axonal conduction, is altered in NCX3^{HOM} mice (Related to Figure 3,4 and 6) **A.** The Rheobase of small DRG neurons was significantly reduced in NCX3^{HOM} mice. (WT n= 35, NCX3^{HOM} n = 29, Mann-Whitney test, *P<0.05). **B.** Fewer small DRG neurons repetitively fire APs in response to 500ms current injections ranging 0-950 pA. (WT n=31 rep, 4 single, NCX3^{HOM} n=18 rep, 11 single, Fisher's exact test, *P<0.05). Only repetitively firing neurons were plotted for each genotype. **C.** Repetitively firing small neurons from NCX3^{HOM} mice fire more APs in response to current injections ranging 0-300 pA (WT n=31, NCX3^{HOM} n= 18, RM two-way ANOVA, with Bonferroni's post hoc test, *P<0.05). **D.** Repetitively firing small neurons from NCX3^{HOM} mice fire fewer APs in response to current injections ranging 350-950 pA (WT n=31, NCX3^{HOM} n= 18, RM two-way ANOVA, with Bonferroni's post hoc test, *P<0.05). **E.** The Rheobase of medium DRG neurons was unchanged in NCX3^{HOM} compared to WT mice. (WT n= 33, NCX3^{HOM} n = 32, Mann-Whitney test, n.s P>0.05). **F.** The number of repetitively firing medium sized neuron was unchanged between NCX3^{HOM} and WT mice (WT n=7 rep, 26 single, NCX3^{HOM} n=13 rep, 19 single, Fisher's exact test, P>0.05). All medium neurons were plotted for each genotype. NCX3^{HOM} medium DRG neurons fire more APs in response to 500ms current injections ranging 0-950 pA (WT n=33, NCX3^{HOM} n= 32, RM two-way ANOVA, *P<0.05). **G.** The Rheobase of large DRG neurons was significantly reduced in NCX3^{HOM} mice compared to W**T** (WT n= 14, NCX3^{HOM} n = 13, Mann-Whitney test, ****P<0.0001). **H.** NCX3^{HOM} large DRG neurons fire more APs in response to 500ms current injections ranging 0-950 pA. Note that large WT do not fire APs to current injections below 1000 pA (WT n=14, NCX3^{HOM} n= 13, RM two-way ANOVA, with Bonferroni's post hoc test, *P<0.05, ***P<0.001). **I.** Example traces of a small WT and small NCX3^{HOM} DRG neuron. Traces illustrate APs elicited by 100 and 750 pA. Peak membrane potential at 500ms was analysed in subsequent figures (Circled). **J-L.** The membrane potential at 500ms across increasing current injections for small, medium, and large DRG neurons, respectively. For all cell sizes, NCX3^{HOM} DRG neurons became significantly more depolarised in response to prolonged (500ms) current injections, compared to WT recordings. Note this is most striking for small cells (J) where membrane potential depolarisation exceeds 0mV. (J-L: Mixed Model-effects RM ANOVA with Bonferroni's post hoc test, *P<0.05, **P<0.01, ***P<0.001, **** P<0.0001). Compound action potentials (CAPs) were recorded from mouse saphenous nerves. **M.** A-fibre CAP amplitudes were comparable between WT and NCX3^{HOM} mice (WT n = 5 nerves, 3 mice, NCX3^{HOM} n = 4 nerves, 3 mice, unpaired T-test, P>0.05). **N.** C-fibre CAP amplitudes were comparable between WT and NCX3^{HOM} mice (WT n = 6 nerves, 3 mice, NCX3^{HOM} n = 5 nerves, 3 mice, unpaired T-test, P>0.05). **O.** There was no significant difference in the C-fibre CAP conduction velocities of WT or NCX3^{HOM} nerves (WT n = 6 nerves, 3 mice, NCX3^{HOM} n = 5 nerves, 3 mice, unpaired T-test, P>0.05). **P.** C-CAPs were recorded at 0.25Hz for 16 stimuli. The change in conduction velocity was calculated (Δ%) for the 16th vs 1st stimulus. C-CAPs from WT and NCX3^{HOM} nerves did not exhibit C/V slowing during this protocol. **Q.** C-CAPs were recorded at 2Hz for 16 stimuli. The change in conduction velocity was calculated (Δ%) for the 16th vs 1st stimulus. C-CAPs from WT and NCX3^{HOM} nerves exhibited very little C/V slowing during this protocol. **R.** C-CAPs were recorded at 2Hz for 240 stimuli. The change in conduction velocity was calculated (Δ%) for the 240th vs 1st stimulus. C-CAPs from both WT and NCX3^{HOM} nerves exhibited C/V slowing during this protocol. P-R, no differences were observed between

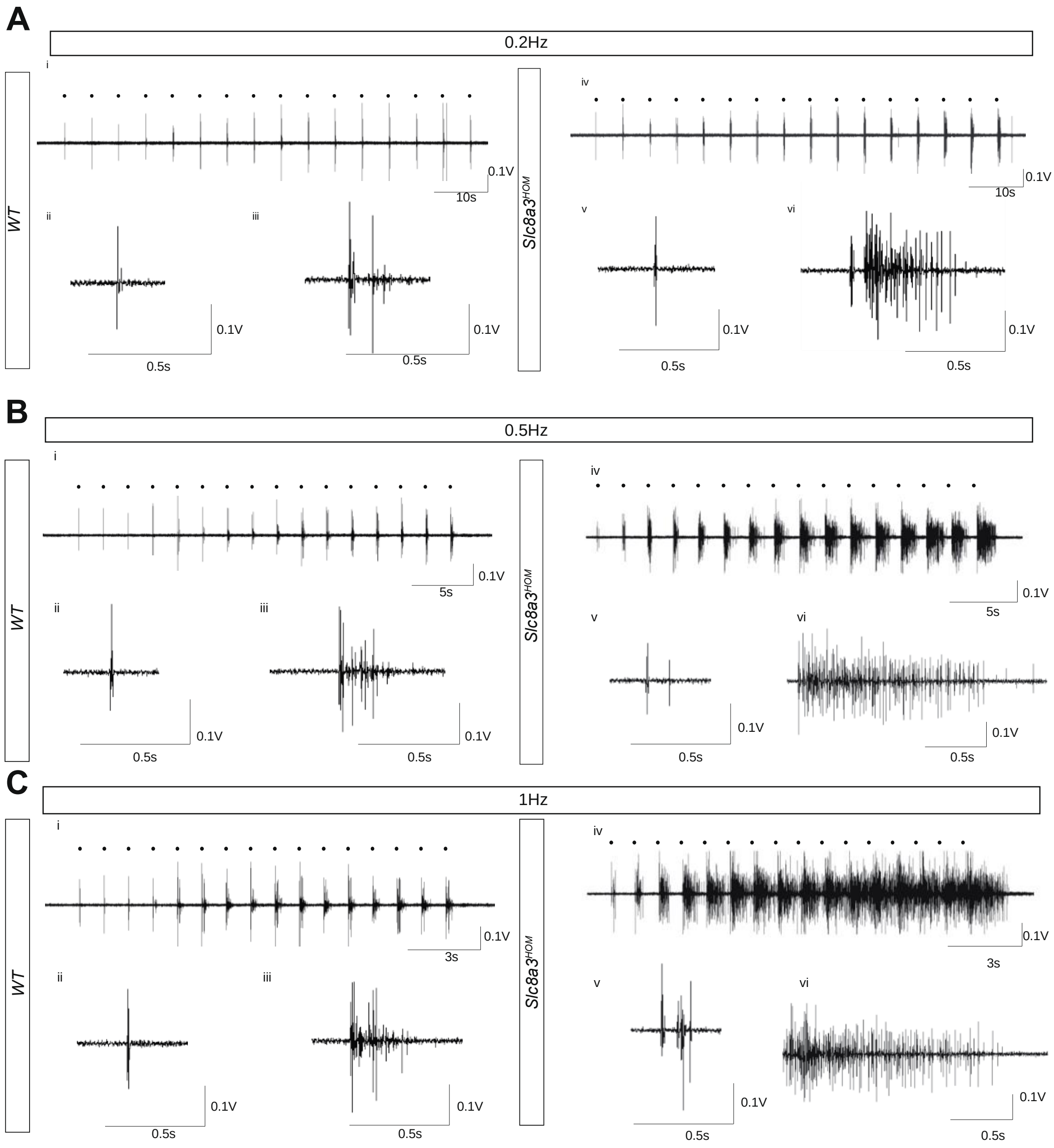
A
0.2Hz
WT
Slc8a3HOM
i
ii
iii
iv
v
vi
0.1V
10s
0.5s
B
0.5Hz
5s
C
1Hz
3s

Supplementary Figure 7. Enhanced flexion reflex motoneuron responses. (Related to Figure 7.E) **A-C.** Example traces at 0.2, 0.5 and 1Hz stimulation for WT (left panels) and $NCX3^{HOM}$ mice (right panels), during the train of 16 stimulations (i and iv), at stimulus 1 (ii and v) or at stimulus 16 (iii or vi)

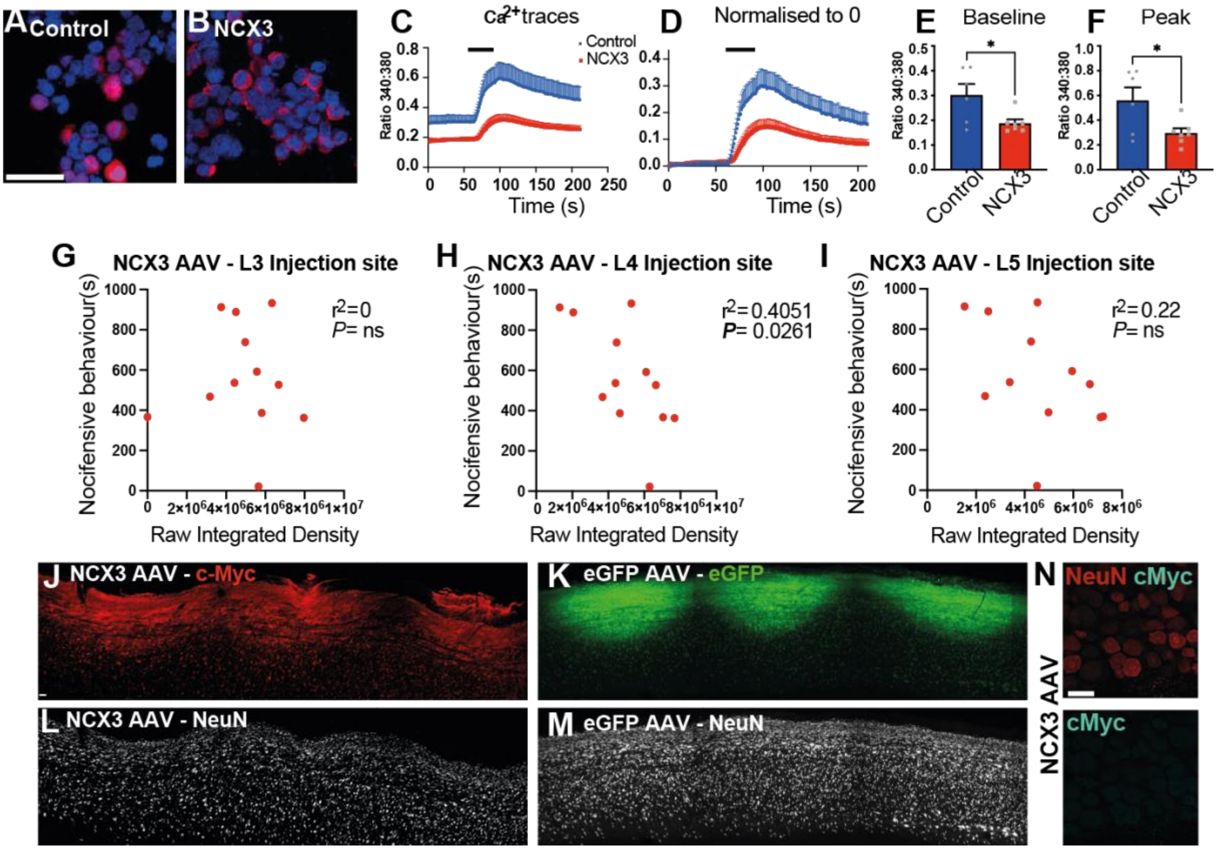
A Control
B NCX3
C
Ca2+ traces
Control
NCX3
Ratio 340:380
Time (s)
D
Normalised to 0
Time (s)
E
Baseline
Ratio 340:380
Control
NCX3
F
Peak
Ratio 340:380
Control
NCX3
G
NCX3 AAV - L3 Injection site
Nocifensive behaviour(s)
Raw Integrated Density
r2=0
P= ns
H
NCX3 AAV - L4 Injection site
Nocifensive behaviour(s)
Raw Integrated Density
r2=0.4051
P= 0.0261
I
NCX3 AAV - L5 Injection site
Nocifensive behaviour(s)
Raw Integrated Density
r2=0.22
P= ns
J NCX3 AAV - c-Myc
K eGFP AAV - eGFP
L NCX3 AAV - NeuN
M eGFP AAV - NeuN
N
NeuN cMyc
cMyc
NCX3 AAV

Supplementary Figure 8. Overexpression of NCX3 – HEK cells and *in vivo* characteristics. (Related to Figure 8) **A and B.** Expression of mCherry (control) and NCX3-B-c-Myc plasmids in HEK cells. **C and D.** Ca^{2+} traces during 1µM Ionomycin treatment (mean ± SEM). Black bar shows duration of treatment. **E and F.** Baseline and peak quantification of traces. **G-I.** Pearson's correlation between nocifensive behaviour in the *NCX3*-B-injected group during the second phase of the formalin test and raw integrated density (collective measure of size and staining intensity at the injection site). Note that at the level of L4 there was a significant negative correlation between the duration of nocifensive behaviour and the density of c-Myc immunostaining. **J-M.** Expression of *NCX3*-B-c-Myc and eGFP in spinal cord sagittal sections at L3, 4, 5 spinal segments. **N.** Representative images of DRG sections post-surgery. Note that no c-myc positive cells were detected in the DRG. Ca^{2+} imaging statistics (E and F) – * $P<0.05$, unpaired Student's T-test (n=6 Control and n=7 NCX3 coverslips). Pearson's correlation (G-I) calculated by Graph Pad Prism 9.0. Statistics - two-tailed statistical test as recommended by software. Scale bars = 50 µm.
